# Granulocyte Colony Stimulating Factor and Physiotherapy after Stroke: Results of a Feasibility Randomised Controlled Trial: Stem Cell Trial of Recovery EnhanceMent after Stroke-3 (STEMS-3 ISRCTN16714730)

**DOI:** 10.1371/journal.pone.0161359

**Published:** 2016-09-09

**Authors:** Nikola Sprigg, Rebecca O’Connor, Lisa Woodhouse, Kailash Krishnan, Timothy J. England, Louise A. Connell, Marion F. Walker, Philip M. Bath

**Affiliations:** 1 Stroke Trials Unit, Division of Clinical Neuroscience, University of Nottingham, Nottingham, United Kingdom; 2 Division of Medical Sciences and GEM, University of Nottingham, Nottingham, United Kingdom; 3 Division of Rehabilitation and Ageing, University of Nottingham, Nottingham, United Kingdom; 4 School of Health, University of Central Lancashire, Preston, United Kingdom; University of Glasgow, UNITED KINGDOM

## Abstract

**Background:**

Granulocyte-colony stimulating factor (G-CSF) mobilises endogenous haematopoietic stem cells and enhances recovery in experimental stroke. Recovery may also be dependent on an enriched environment and physical activity. G-CSF may have the potential to enhance recovery when used in combination with physiotherapy, in patients with disability late after stroke.

**Methods:**

A pilot 2 x 2 factorial randomised (1:1) placebo-controlled trial of G-CSF (double-blind), and/or a 6 week course of physiotherapy, in 60 participants with disability (mRS >1), at least 3 months after stroke. Primary outcome was feasibility, acceptability and tolerability. Secondary outcomes included death, dependency, motor function and quality of life measured 90 and 365 days after enrolment.

**Results:**

Recruitment to the trial was feasible and acceptable; of 118 screened patients, 92 were eligible and 32 declined to participate. 60 patients were recruited between November 2011 and July 2013. All participants received some allocated treatment. Although 29 out of 30 participants received all 5 G-CSF/placebo injections, only 7 of 30 participants received all 18 therapy sessions. G-CSF was well tolerated but associated with a tendency to more adverse events than placebo (16 vs 10 patients, p = 0.12) and serious adverse events (SAE) (9 vs 3, p = 0.10). On average, patients received 14 (out of 18 planned) therapy sessions, interquartile range [12, 17]. Only a minority (23%) of participants completed all physiotherapy sessions, a large proportion of sessions (114 of 540, 21%) were cancelled due to patient (94, 17%) and therapist factors (20, 4%). No significant differences in functional outcomes were detected in either the G-CSF or physiotherapy group at day 90 or 365.

**Conclusions:**

Delivery of G-CSF is feasible in chronic stroke. However, the study failed to demonstrate feasibility for delivering additional physiotherapy sessions late after stroke therefore a definitive study using this trial design is not supported. Future work should occur earlier after stroke, alongside on-going clinical rehabilitation.

**Trial Registration:**

ISRCTN.com ISRCTN16714730

## Introduction

Stroke is the second leading cause of disability worldwide, with half of survivors being dependent on other people six months later[1]. The incidence of stroke increases almost exponentially with age and, in combination with an aging population, the burden of stroke to survivors, their families and society is increasing.

Rehabilitation is thought to promote functional recovery through neuroplastic changes and evidence suggests plasticity extends beyond the sub-acute stage.[2] Despite this, the majority of patients do not receive rehabilitation therapy beyond three months, although some receive community input for up to six months post stroke. The dose, method and intensity of therapy appear to be important and most effective when delivered as high intensity task specific practice.[3] Therapy at home may be beneficial,[4] but the long term benefit of therapy in chronic stroke is not known.[5] In experimental stroke, ‘top-up’ bursts of therapy improve functional outcome[6] but this has not been demonstrated in clinical studies.

Pharmacological agents may increase the benefit of intensive therapy.[7] Potential therapeutic options for pharmacological enhancement of recovery include neurochemical approaches, using agents such as amphetamines,[8] or SSRI’s.[9] Another approach is a neuroreparative paradigm, using agents such as stem cells, or agents that release endogenous stem cells.

Granulocyte Colony Stimulating Factor (G-CSF) mobilises endogenous haematopoietic (CD34+) bone marrow stem cells into the circulation and is routinely used in stem cell transplantation in haematological malignancy.[10] G-CSF has a multi-modal action that has the potential to be both neuroprotective (anti-apoptotic) and neuroreparative, the latter through mechanisms that include stem cell mobilisation, neurogenesis and angiogenesis. In experimental models of stroke, when given soon after infarct, G-CSF improves recovery.[11, 12] In clinical stroke, G-CSF when given in similar doses as used in haematology, was effective at mobilising CD34+ stem cells[13] and a number of trials have since been completed in acute[14, 15] and sub acute stroke.[16] In the largest study,[15] G-CSF showed no evidence of efficacy in 328 patients with hyperacute stroke. However, G-CSF was given intravenously and unexpected haemodynamic effects were reported potentially counteracting any potential beneficial effects of G-CSF. Meta-analysis of all studies to date showed a non-significant reduction in early impairment but no effect on functional outcome.[17]

In an experimental model of later administration, G-CSF when given with Stem cell factor improved outcome even when given late after infarct.[18] To date, in one small clinical study of G-CSF in chronic stroke, G-CSF was tolerable but no beneficial effect was seen on outcome, although its administration was not combined with rehabilitation therapy.[19]

In experimental stroke, pharmacological augmentation of recovery is dependent on enriched environment and stimulation–hence the rationale for combining G-CSF and physiotherapy.[20] We hypothesised that in chronic stroke intensive therapy can stimulate use dependent plasticity, which may be augmented by G-CSF. The factorial design of this pilot randomised controlled trial tests the feasibility of investigating this theory further.

## Methods

We performed a prospective blinded-outcome, single-centre, two-by-two factorial randomised (1:1) placebo-controlled trial of G-CSF (Filgastrim, 1x10E6 iu/kg) and/or rehabilitation therapy in 60 adults three months to two years post stroke. Participants had to demonstrate residual disability (modified Rankin scale, mRS >1) and no longer be receiving on-going rehabilitation. Exclusion criteria were inability to provide informed consent; lack of residual motor deficit; significant cognitive impairment that will impede ability to complete assessments; diagnosis likely to interfere with outcome or rehabilitation (e.g. terminal illness); still receiving post stroke rehabilitation; pregnancy; and other exclusions of G-CSF as listed in the British National Formulary. Potentially eligible patients were identified by clinical staff from Nottingham University Hospital stroke service, NHS Nottingham City and NHS Nottinghamshire County community stroke teams, who sought verbal permission from the patient or representative to be screened for eligibility for the study. Eligible patients were then approached for participation by the research team. All participants provided written informed consent.

The study was approved by the Yorkshire and Humber Research Committee (11/YH/0138, 20/06/2011), had a Medicines and Healthcare products Regulatory Agency Clinical Trial Authorisation (13/05/2011-11015). The study was prospectively submitted for ISRCTN registration on 28/07/2015 before the recruitment of the first participant. Following this submission, there were no subsequent changes to the information as supplied in the study record. Study recruitment started on 01/11/2011. Registration ISRCTN16714730 occurred on 8^th^ December after the NIHR confirmed the study had been adopted to NIHR portfolio. The study was performed according to the Declaration of Helsinki and the International Conference on Harmonisation of Good Clinical Practice.

### Funding

The study was funded by National Institute Health Research, Research for Patient Benefit (RfPB) Grant.

### Randomisation

Randomisation was performed using minimisation to reduce baseline imbalances; minimisation was performed on: age; gender; time from stroke; motor function (Rivermead Motor Assessment, RMA. In 5% of randomisations, assignment was reversed so as to reduce predictability. Drug treatment allocation was concealed to all research staff and participants throughout the study. Only the treating research therapist and participant knew the therapy group allocation.

### Intervention

**Fig 1:**
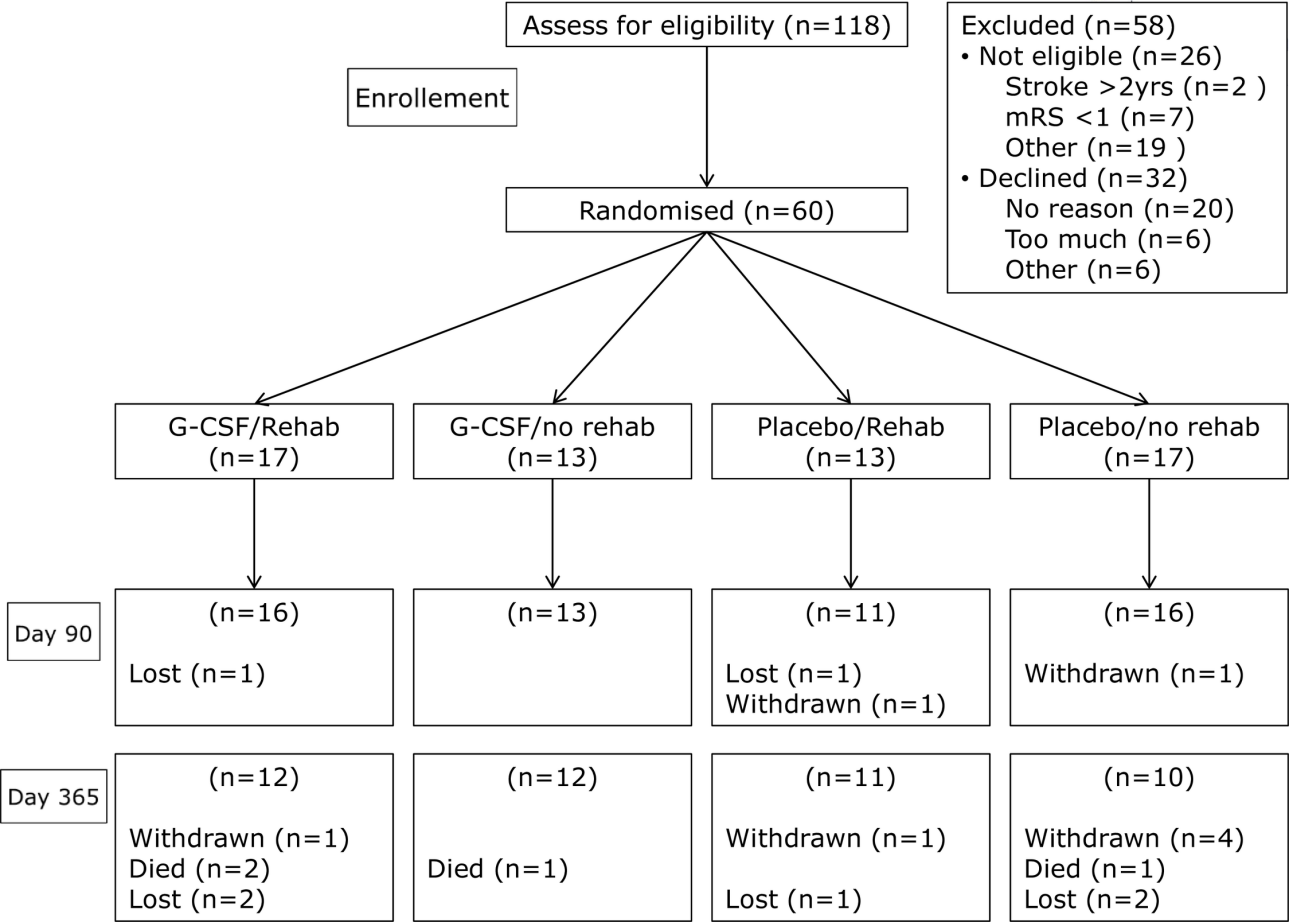
Recruitment flow chart.

Participants received a subcutaneous injection once/day on 5 consecutive days at home of either G-CSF or placebo (normal saline 0.9%) in identical syringes (administered by a research nurse) and a rehabilitation intervention (of 18 home-based therapy sessions) or standard care (no additional rehabilitation) (Fig 1). Dosing of both interventions was based on previous research, with G-CSF after stroke,[13] and evidence that 17 hours of therapy improved outcome after stroke.[21]

Those randomised to the rehabilitation intervention were allocated to receive 18 sessions 45 minutes long, 3 times a week, for 6 weeks. Rehabilitation was delivered by a research physiotherapist using a patient orientated goal approach, tailored to the patient’s individual needs, with sessions at a time of patients choosing. Where appropriate, independent exercise programmes were issued for the patient to continue rehabilitation in their own time, if they wished to do so, as would occur in routine practice. Carers were also encouraged to be involved in rehabilitation where appropriate. Those randomised to the standard card intervention received no additional therapy.

The primary outcomes were feasibility (proportion of participants receiving all 5 G-CSF/placebo injections, proportion of participants receiving all therapy sessions); acceptability (proportion of participants screened who are eligible for enrolment who give consent); and tolerability -adverse events (headache, backache, flu like symptoms) after drug administration, proportion of participants who decline rehabilitation therapy sessions.

Secondary outcomes included haematological measures (Day 5 CD34+ count, full blood count, platelet count); safety; end of follow-up (Day 90) death (cause), stroke recurrence, infection and serious adverse events (SAE). Clinical exploratory efficacy measures were performed face to face at baseline, Day 45, Day 90, Day 365 and included motor function (Rivermead Motor Assessment, RMA)[22]; dependency (mRS) shift)[23]; disability (change in Barthel Index, BI)[24]; quality of life (EuroQoL)[25]; Nottingham extended activities of daily living (NEADL)[26], Mini-mental state examination (MMSE)[27], mood (Zung)[28] and care giver burden (GHQ-28)[29]. All follow-up assessments were performed blinded to treatment allocation and laboratory results. Serious adverse events were reviewed independently, blinded to treatment allocation and categorised using standard definitions.[30]

### Statistics

As a pilot study assessing feasibility, no formal sample size calculation was performed. A sample size of 60 was deemed *a priori* to be adequate to test the feasibility and safety of the interventions; the sample size was not designed to test formally efficacy or interaction between the interventions. The primary and secondary outcomes were compared between participants randomised to G-CSF versus placebo (intention-to-treat) and therapy versus control. Comparisons between groups are by Chi-Square, t-test or ANOVA and a p value < 0.05 is considered significant. Interaction between G-CSF and therapy is tested by ordinal logistic regression (using mRS score at day 90 as the response variable and G-CSF, Therapy, G-CSF*Therapy [interaction term], Age, Sex, mRS and NIHSS scores at baseline and time from stroke to randomisation as covariates). Analyses were performed using SAS version 9.3. No corrections for multiple statistical tests were performed.

## Results

60 participants were recruited between 21^st^ November 2011 and July 2013. The recruitment flow chart is shown in Fig 1. Last patient visit was 21st May 2014. The baseline characteristics were well matched across the treatment groups (Table 1), mean age 66.2 years, total anterior circulation stroke syndrome 45%, recruited at a mean of 347 days after stroke, with residual dependency (mean mRS 3.1).

**Table 1:** Baseline characteristics by treatment groups. Data are shown as mean (standard deviation) and number (%).

Baseline Factor	G-CSF	G-CSF	Placebo	Placebo	All
	Rehab	No rehab	Rehab	No rehab	
	N = 17	N = 13	N = 13	N = 17	N = 60
Age*, mean (SD)	66.8 (8.1)	66.8 (9.0)	64.0 (11.2)	66.9 (14.1)	66.2 (10.7)
Male*, n (%)	11 (65)	6 (46)	8 (62)	11 (65)	36 (60)
Time since stroke* (days)	347.9 (138.0)	335.1 (141.4)	348.2 (136.1)	355.0 (152.5)	347.2 (139.1)
mRS, mean (SD)	3.2 (0.95)	3.2 (0.99)	3.1 (0.95)	3.1 (0.86)	3.1 (0.91)
RMA*, mean (SD)	16.9 (11.2)	18.8 (9.3)	19.2 (9.5)	16.9 (8.2)	17.8 (9.5)
NIHSS, mean (SD)	7.2 (5.3)	6.1 (4.0)	5.6 (3.8)	6.2 (3.9)	6.3 (4.3)
TACS n (%)	8 (47)	6 (46)	5 (38)	8 (47)	27 (45)
PACS n (%)	4 (24)	4 (31)	3 (23)	3 (18)	14 (23)
LACS n (%)	4 (24)	1 (8)	4 (31)	4 (24)	13 (22)
Prior stroke, n (%)	7 (41)	3 (23)	3 (23)	2 (12)	15 (25)
History TIA, n (%)	3 (18)	2 (15)	1 (8)	4 (24)	10 (17)
Hypertension, n (%)	15 (88)	10 (77)	8 (62)	14 (82)	47 (78)
History AF, n (%)	5 (29)	3 (23)	3 (23)	6 (35)	17 (28)
History diabetes, n (%)	5 (29)	6 (46)	4 (31)	6 (35)	21 (35)

* Minimisation data; mean (standard deviation SD) mRS modified Rankin scale; RMA, Rivermead motor assessment; NIHSS National Institutes of Health Stroke Scale; TACS total anterior circulation syndrome; PACS partial anterior circulation syndrome; LACS lacunar stroke syndrome; TIA transient ischaemic attack, AF atrial fibrillation.

Primary outcome: Recruitment to the trial was feasible and acceptable; with 60 participants recruited (out of 118 screened). 32 out of 92 declined to participate. All participants received some of their allocated treatment.

### G-CSF/placebo administration

Feasibility: All patients received their randomised treatment allocation. 29 out of 30 participants received all 5 injections; 1 patient in the G-CSF group discontinued the treatment after 4 days due to an adverse event (headache).

Tolerability: More patients reported adverse events in the G-CSF group (Table D in S1 file). Similarly, more patients in the G-CSF group (n = 9) reported serious adverse events than in the control group (3), although this was not statistically significant (p = 0.10) (Table 2).

**Table 2:** Serious Adverse Events, by group and time to event in days; includes all SAEs up to day 90, and any fatal SAEs post day 90.

	Serious Adverse Events			
Time to event	Group A	Group B	Group C	Group D
	GCSF and Therapy N = 17	GCSF and No therapy N = 13	No GCSF and Therapy N = 13	No GCSF No Therapy N = 17
**Immediate *≤ 14 days***	UTI	Hyperglycaemia Neutrophilia		
**Early *15–42 days***	Shingles	Ischaemic stroke	UTI	Seizure
	Cellulitis			
**Late *43–90 days***	Ischaemic stroke, Muscle Cramps, Fracture neck femur	Seizure, Overdose		Ischaemic stroke
**Fatal SAEs (days after treatment)**	Fracture neck femur (244), Peripheral vascular disease (322)	Pneumonia (306)		Cardiac failure (129)

**Fig 2:**
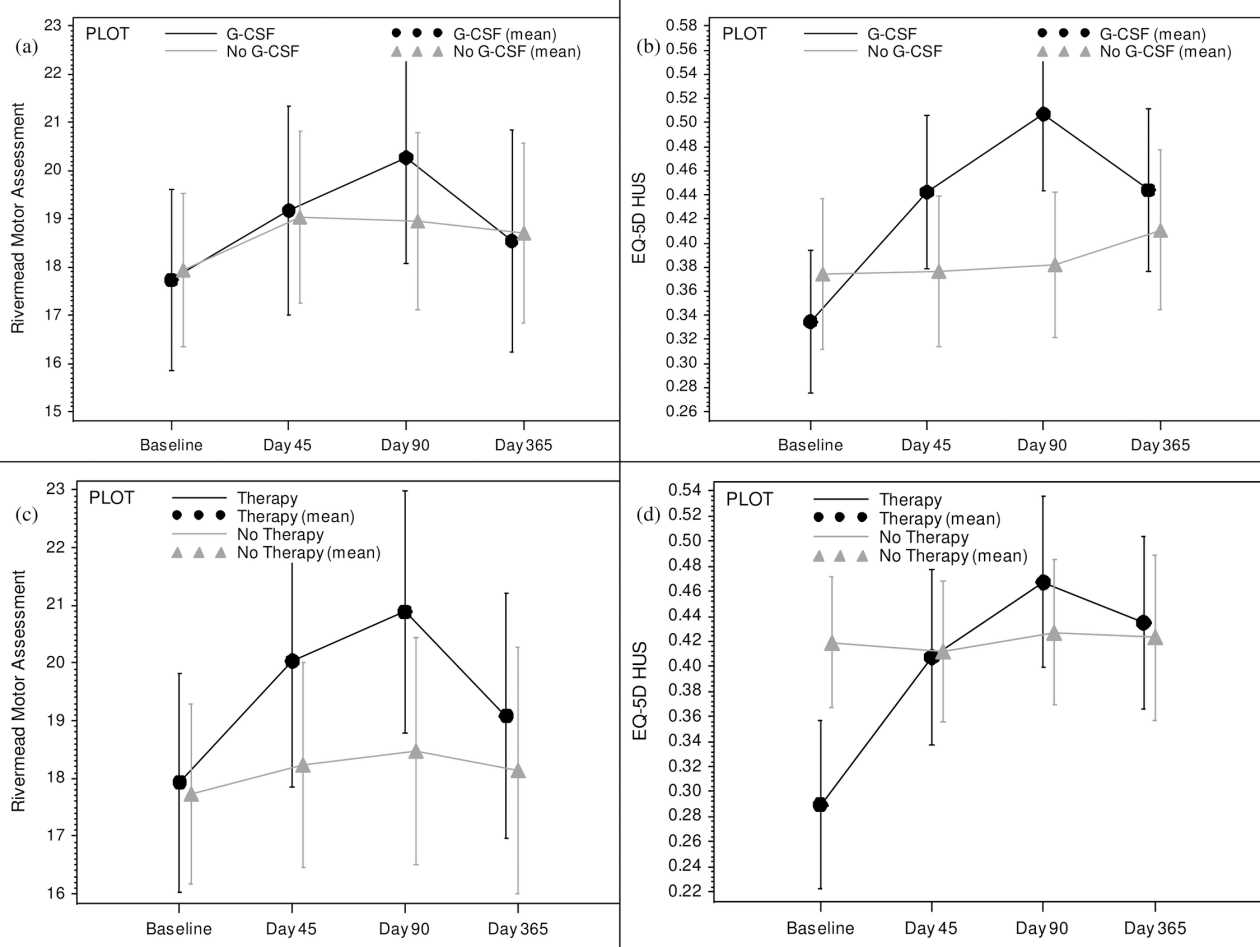
Recovery outcome measures by treatment group (bars represent the standard error). a) Rivermead Motor Assessment (RMA) by G-CSF vs. placebo. b) Quality of life (EQ-5D) by G-CSF vs. placebo. c) Rivermead Motor Assessment (RMA) by physiotherapy vs. control. d) Quality of life (EQ-5D) by physiotherapy vs. control.

Functional Outcomes: There was no significant difference in dependency or disability outcomes between the G-CSF and control treatment groups. In univariate analysis, using a t-test to compare the mean differences, there was significant improvement in EQ-5D QoL in the G-CSF group (Table 3, Fig 2).

**Table 3:** Treatment group comparison of the mean difference in outcome between baseline and day 90; G-CSF vs. No G-CSF and Therapy vs. No therapy.

Outcome	G-CSF	No G-CSF N = 30	2p	Therapy N = 30	No Therapy N = 30	2p
	**N = 30**					
Modified Rankin scale (mRS)	-0.37	-0.22	0.46	-0.29	-0.31	0.90
NIHSS	-0.93	-0.96	0.96	-0.88	-1	0.88
Barthel index (BI)	-2.76	-3.33	0.87	-3.33	-2.76	0.87
Rivermead motor assessment (RMA)	2.03	0.81	0.23	2.37	0.59	0.072
Berg balance scale (BBS)	2.52	-0.48	0.18	2.96	-0.69	0.091
EuroQoL-5D	0.15	-0.02	**0.048**	0.12	0.02	0.26
EuroQoL VAS	11.12	8.48	0.66	8.1	11.48	0.57
Zung depression scale	-2.39	-0.15	0.37	-2	-0.61	0.58
NE-ADL	3.48	0.22	0.39	4.3	-0.31	0.22
Mini-mental state examination (MMSE)	1.07	0.96	0.92	0.81	1.21	0.69
Carer giver burden: (GHQ-28)	0.5	10.44	0.14	3.91	5.7	0.81

National Institutes of Health Stroke Scale (NIHSS); Nottingham extended activities of daily living (NE-ADL)general health questionnaire (GHQ-28).

This remained significant following adjustment for Age, Sex, mRS and NIHSS scores at baseline and time from stroke to randomisation as covariates.(p = 0.037)

Haematological outcomes: G-CSF significantly increased white cell and CD34 counts, demonstrating that it was effective at mobilising haematopoietic stem cells. There were no effects on red cell or platelet count (Table B in S1 File).

### Rehabilitation/no rehabilitation

Feasibility: All patients received their randomised treatment allocation. Only 7 (23%) participants received all 18 sessions; just over half, 16 (53%), missed more than 3 sessions. On average patients received 14 therapy sessions, (interquartile range 12 to 17). The distribution of therapy sessions received by patients overall and by group is shown in Figs A, B and C in S2 File. The most common reason for missing a therapy session was patient unavailability (illness: 62 sessions; social/family commitments: 22 sessions; medical appointment: 8 sessions; fall: 2 sessions). A number of therapy sessions (4%) were also missed due to therapist availability. There were no significant differences in age, gender and stroke severity between those that had all their therapy sessions versus those that did not (Table C in S1 File).

Tolerability: There were 6 patients each with serious adverse events in the rehabilitation and control groups (Table 2). Patients in the rehabilitation group were less likely to report falls at day 45 and day 90 than those in the no rehabilitation group (day 45: 23.3% vs. 33.3%, p = 0.57; and day 90: 13% vs. 30%, p = 0.20) but this was not statistically significant (Table D in S1 File).

Functional Outcomes: There were no significant differences in outcomes between the therapy and no therapy groups, and no consistent trends in change from baseline to day 90 and day 365. (Table 3) Patients in the therapy group showed improvement in Rivermead Motor Assessment at end of therapy (Day 45), but this was not statistically significant (Fig 2).

Haematological outcomes: There were no effect on haematological measures between the therapy and no therapy groups. Platelet count was statistically different between the groups, but this was present at baseline and not clinically significant (Table B in S1 File).

Interaction between interventions: In multivariate ordinal logistic regression, there was no significant evidence of an interaction between the two interventions G -CSF and therapy (OR 1.20, 95% CI [0.14, 10.64], p = 0.87). The only significant predictor of outcome was baseline function assessed as the mRS.

### Data completion: Fig 1

Day 90: No participants died during the 90 day follow up period. 2 participants withdrew from the trial before day 90–1 was in the no therapy group and expressed disappointment at not receiving therapy. 2 participants were lost to follow-up (1 G-CSF and therapy group, 1 placebo and therapy) by day 90.

Day 365: 3 participants died in the G-CSF group and 1 in the placebo group. (Table 2) More participants were either lost or withdrawn at day 365 (total 5 lost, 6 withdrawn) compared to day 90 (2 withdrawn). Four of the six withdrawn participants were randomised to no physiotherapy.

Consort checklist (S1 Checklist) and CTIMP protocol (S1 Protocol) are provided as supporting information files.

## Discussion

In this trial, using a factorial design, we have demonstrated that it is feasible to enrol participants with chronic stroke from the community. Recruitment was as planned and potential participants straightforward to identify. Two thirds of potentially eligible participants chose to participate, higher than seen in other studies.[31] This perhaps reflects both the burden of need amongst stroke survivors and their carers, and willingness to explore the potential of drugs to improve outcome after stroke.[32] It was feasible to deliver the drug G-CSF in the community, and G-CSF was, on the whole, well tolerated. However, not all participants randomised to physiotherapy received the planned quantity of sessions. This was despite the therapist being flexible regarding timing of therapy sessions. Anecdotally, participants and their clinicians reported that they found randomisation to no additional physiotherapy less acceptable. This is being explored in a qualitative sub-study that is being prepared for publication.[33]

Exploratory measures of efficacy showed no differences in outcome in the G-CSF group, although the trial was not powered to test outcome and a difference cannot be excluded. Although there was significant improvement in quality of life this finding could be due to chance, as we did not perform multiple statistical tests. The only other study of G-CSF in chronic stroke included participants with very minor residual motor deficit. Although trends to improved outcomes were demonstrated with G-CSF, methodological issues limit interpretation of this study.[19] Any potential benefit in chronic stroke would be in contrast to results in hyperacute stroke. In the large AXIS-2 study,[34] G-CSF showed no evidence of efficacy in 328 patients with hyperacute stroke. However, AXIS-2, was testing a neuroprotective paradigm, rather than the neuroreparative one tested here. Furthermore, recent experimental data has shown that the dose used in AXIS-2 is likely to have lead to significant immunomodulation, with possible modulation of T cell response and dendritic cells,[35] the safety consequences of which are unknown in acute stroke.

This study provides more safety data on the use of G-CSF after stroke. Despite an expected increase in reported adverse events, all but one participant tolerated a full 5 day course of G-CSF. As seen in previous stroke studies, there were more SAEs in the G-CSF group, and some of these (infections, vascular events) were possibly related to G-CSF treatment. This is in keeping with data from haematology and oncology studies over many decades, where G-CSF was well tolerated,[36] and side effects of G-CSF treatment are self-limiting and clinically acceptable.[37]

The rehabilitation intervention was safe and there were no increase in falls or SAEs. Participants allocated to therapy did show improvement in function at day 45 (end-of-treatment) although this was not statistically significant at end-of-follow up. However, less than a quarter of patients received all of their allocated therapy sessions, predominantly due to patient factors rather than therapist availability. There was no difference in therapy session delivery between the G-CSF group and the placebo group. The feasibility of delivering such a therapy intervention (3 sessions/week) so long after stroke therefore appears to be sub-optimal. This lower-than-expected uptake of therapy sessions is in contrast to some other research studies,[38, 39] and may relate to a number of factors including type of participants (with varying deficits) and limited staff (with only one physiotherapist available). However another therapy intervention delivered to chronic stroke survivors has also shown limited uptake of sessions. [40] This may reflect clinical practice, where in reality many patients miss sessions for a variety of reasons.

Assessing fidelity of the therapy intervention also needs to capture more subjective factors such as content of therapy, patient motivation and patient relationship with the therapist. For instance, the number of sessions attended or length of therapy session may not be the best way to record intensity.[31] These factors would be best explored through qualitative research, beyond the scope of the present work.

Feedback from participants highlighted a number of issues with the trial design. In order to allow for a control therapy group we deliberately only included patients who had already been discharged from therapy prior to enrolment. Despite undergoing a comprehensive informed consent process, some patients were unsurprisingly disappointed to not receive additional therapy. This led to selection bias when clinicians recognised that emotional distress caused by randomisation to the control (no physical therapy) group would not be well tolerated and did not recommend patients for participation in the trial. Hence, this design may not be appropriate for future studies, where it may be preferable to deliver G-CSF alongside on-going rehabilitation. We did not deliver a cross over design as this would have precluded long term follow-up, which is critical when measuring recovery after stroke.

This study has a number of limitations; foremost this was a feasibility study performed in a single centre, and experience of participants may not reflect those seen in other centres. Furthermore, a heterogeneous group of participants was included, and this may limit potential to detect efficacy. Animal data is based on models of large artery occlusion, as seen clinically in cortical infarction–there is no experimental data in lacunar stroke or small vessel disease.[41] However, the only clinical study of G-CSF in chronic stroke was performed in elderly patients with white matter disease.[19] When this study was developed in conjunction with stroke survivors, they strongly argued for broad inclusion criteria, to make the study more accessible and the results generalizable. Whilst this is important in a definitive phase 3 study, it may have compromised the likelihood of achieving interpretable findings in this phase 2 study. Unfortunately study funding did not permit multi-disciplinary rehabilitation therapy input so physiotherapy was chosen to focus on motor recovery. We did however explore efficacy in other domains including cognition, mood and quality of life. A number of studies have recently shown significant differences in the outcome of patient reported quality of life,[42] as we did here with G-CSF. This finding warrants further exploration. Finally, the timing and duration of the therapy intervention in this population may have been too little too late. Experimental models showing that plasticity is greatest earlier after stroke,[7] and that recovery is related to use-dependent.[43] Therapy so late after stroke may be better aimed at preventing deterioration, rather than stimulating plasticity. Future work should focus on participants where stimulating plasticity is more like, earlier after stroke.

In summary, this is the first study to assess the feasibly of testing the combination of G-CSF and physiotherapy in chronic stroke. Whilst delivery of G-CSF in chronic stroke was feasible and well tolerated, the acceptability of being randomised to no physiotherapy was a limiting factor, and we failed to achieve the planned quantity of additional therapy. As such, we believe larger factorial trials to study the combination of G-SF and physiotherapy using this design are not supported. Future work should focus on safety and efficacy of G-CSF given earlier after stroke onset, alongside on-going clinical rehabilitation, to stimulate use dependent plasticity.

## Supporting Information

S1 Checklist Consort Checklist: CONSORT 2010 checklist of information to include when reporting a randomised trial. (DOC)

S1 File Additional result tables. (A) Side effects reported by drug treatment group. (B) Blood counts at day 5 by treatment groups. (C) Comparison of baseline factors between participants who had 15–18 sessions with those who had under 15. (D) Falls reported by treatment group. (E) Group comparison of the mean difference between outcomes at baseline and day 90. (F) Treatment group comparison of the mean difference in outcome between baseline and day 365.
(DOCX)

S2 File Figures illustrating number of therapy sessions. (A) Distribution of the number of therapy sessions received by participants randomised to receive physiotherapy. (B) Distribution of the number of therapy sessions received by participants randomised to G-CSF. (C) Distribution of the number of therapy sessions received by participants randomised to No G-CSF.
(DOCX)

S1 Protocol CTIMP Protocol: “Stem cell Trial of recovery EnhanceMent after Stroke 3’ (STEMS 3)- a pilot randomised controlled trial of G-CSF and therapy in chronic stroke. Protocol Version 1.3. 28th August 2012.
(DOC)
