# Supplementary material for: Granulocyte Colony Stimulating Factor and Physiotherapy after Stroke: Results of a Feasibility Randomised Controlled Trial: Stem Cell Trial of Recovery EnhanceMent after Stroke-3 (STEMS-3 ISRCTN16714730)
Source: PLoS One. 2016 Sep 9;11(9):e0161359. doi: 10.1371/journal.pone.0161359 (PMC5017715; doi:10.1371/journal.pone.0161359)
Supplement: S1 File — (A) Side effects reported by drug treatment group. (B) Blood counts at day 5 by treatment groups. (C) Comparison of baseline factors between participants who had 15–18 sessions with those who had under 15. (D) Falls reported by treatment group. (E) Group comparison of the mean difference between outcomes at baseline and day 90. (F) Treatment group comparison of the mean difference in outcome between baseline and day 365. (DOCX) [file pone.0161359.s002.docx]

S1 File Tables

S1 Table A Side effects reported by drug treatment group

S1 Table B Blood counts at day 5 by treatment groups

S1 Table C Comparison of baseline factors between participants who had 15-18 sessions with those who had under 15

S1 Table D Falls reported by treatment group

S1 Table E Group comparison of the mean difference between outcomes at baseline and day 90

S1 Table F Treatment group comparison of the mean difference in outcome between baseline and day 365; G-CSF vs. No G-CSF and Therapy vs. No therapy

**S1 Table A: Side effects reported by treatment group** n (%)

| **Tolerability of drug** | **G-CSF N=30** | **No G-CSF N=30** | **2p** |
| --- | --- | --- | --- |
| Patients with side effect, | 16 (53.3) | 10 (33.3) | 0.12 |
| Bone pain | 6 (20.0) | 3 (10.0) |  |
| Headache | 3 (10.0) | 4 (13.3) |  |
| Infection | 0 (0.0) | 2 (6.7) |  |

**S1 Table B: Blood counts at day 5 by treatment groups. Mean (SD)**

| **Blood parameter** | **G-CSF N=30** | **No G-CSF N=30** | **P-value** |
| --- | --- | --- | --- |
| Haemaglobin (g/dL) | 13.7 (1.50) | 13.7 (1.70) | 0.92 |
| Platelets (×10^9^/L) | 251.0 (79.63) | 253.3 (84.08) | 0.92 |
| White cell count (×10^9^/L) | 36.1 (12.08) | 7.4 (1.55) | <0.0001 |
| CD34^+^ (cells/µl) | 31.1 (21.12) | 3.3 (2.37) | <0.0001 |

**S1 Table C: Comparison of baseline factors between participants who had 15-18 sessions of therapy with those who had under 15. Number (%), Mean (SD)**

| **Variable** | **< 15 sessions** | **15 to 18 sessions** | **2p** |
| --- | --- | --- | --- |
| Gender (Female) | 5 (16.67) | 6 (20.00) | 0.71 |
| Gender (Male) | 11 (36.67) | 8 (26.67) |  |
| Age | 64.25 (9.49) | 67.07 (9.56) | 0.43 |
| NIHSS | 5.31 (4.22) | 7.86 (5.04) | 0.15 |
| mRS | 3.00 (1.03) | 3.29 (0.83) | 0.41 |

**S1 Table D: Falls reported by treatment group**

| **Safety outcome, falls** | **Rehab** | **No rehab** | **2p** |
| --- | --- | --- | --- |
| Reported fall day 45, n (%) | 7 (23.3) | 10 (33.3) | 0.57 |
| Reported fall day 90, n (%) | 4 (13.3) | 9 (30) | 0.20 |

**S1 Table E: Group comparison of the mean difference between outcomes at baseline and day 90**

| **Outcome** | **Group A** GCSF and therapy  N=17 | **Group B** GCSF and No therapy  N=13 | **Group C** No GCSF and Therapy N=13 | **Group D** No GCSF No Therapy N=17 | **2p** |
| --- | --- | --- | --- | --- | --- |
| Modified Rankin scale (mRS) | -0.35 | -0.38 | -0.18 | -0.25 | 0.90 |
| NIHSS | -0.69 | -1.23 | -1.2 | -0.81 | 0.95 |
| Barthel index (BI) | -3.75 | -1.54 | -2.73 | -3.75 | 0.97 |
| Rivermead motor assessment (RMA) | 2.38 | 1.62 | 2.36 | -0.25 | 0.17 |
| Berg balance scale (BBS) | 3.81 | 0.92 | 1.73 | -2 | 0.24 |
| EuroQoL-5D | 0.08 | 0.08 | 0 | -0.15 | 0.15 |
| EuroQoL VAS | 8.91 | 13.85 | 6.91 | 9.56 | 0.89 |
| Zung depression scale | -2.38 | -2.42 | -1.45 | 0.75 | 0.76 |
| NE-ADL | 6.56 | -0.31 | 1 | -0.31 | 0.48 |
| Mini-mental state examination (MMSE) | 0.13 | 2.33 | 1.82 | 0.38 | 0.34 |
| Carer giver burden: (GHQ-28) | -1.83 | 2.83 | 10.8 | 10 | 0.55 |

National Institutes of Health Stroke Scale (NIHSS); Nottingham extended activities of daily living (NE-ADL)
General health questionnaire (GHQ-28)

**S1 Table F: Treatment group comparison of the mean difference in outcome between baseline and day 365; G-CSF vs. No G-CSF and Therapy vs. No therapy**

| **Outcome** | **GCSF N=30** | **No GCSF N=30** | **2p** | **Therapy N=30** | **No Therapy N=30** | **2p** |
| --- | --- | --- | --- | --- | --- | --- |
| Modified Rankin scale (mRS) | -0.07 | -0.14 | 0.81 | -0.16 | -0.04 | 0.64 |
| NIHSS | -0.50 | -1.05 | 0.33 | -0.91 | -0.59 | 0.58 |
| Barthel index (BI) | -5.42 | -2.86 | 0.49 | -6.09 | -2.27 | 0.30 |
| Rivermead motor assessment (RMA) | -0.54 | -0.14 | 0.76 | 0.17 | -0.91 | 0.43 |
| Berg balance scale (BBS) | 0.54 | -0.05 | 0.79 | 1.13 | -0.64 | 0.43 |
| EuroQoL-5D | 0.09 | 0.01 | 0.41 | 0.09 | 0.01 | 0.44 |
| EuroQoL VAS | 4.33 | 7.10 | 0.67 | 8.48 | 2.64 | 0.35 |
| Zung depression scale | -2.45 | -1.43 | 0.72 | -3.13 | -0.60 | 0.37 |
| NE-ADL | -1.42 | -1.38 | 0.99 | -1.17 | -1.64 | 0.90 |
| Mini-mental state examination (MMSE) | 1.48 | -0.57 | 0.11 | 0.26 | 0.76 | 0.70 |
| Carer giver burden: (GHQ-28) | -7.50 | 1.00 | 0.24 | -1.60 | -6.00 | 0.59 |

National Institutes of Health Stroke Scale (NIHSS); Nottingham extended activities of daily living (NE-ADL) general health questionnaire (GHQ-28
