# Supplementary material for: Granulocyte Colony Stimulating Factor and Physiotherapy after Stroke: Results of a Feasibility Randomised Controlled Trial: Stem Cell Trial of Recovery EnhanceMent after Stroke-3 (STEMS-3 ISRCTN16714730)
Source: PLoS One. 2016 Sep 9;11(9):e0161359. doi: 10.1371/journal.pone.0161359 (PMC5017715; doi:10.1371/journal.pone.0161359)

**S2 Fig A,B, C**

**Figure A: Histogram showing the distribution of the number of therapy sessions received by participants randomised to receive physiotherapy (N=30)**


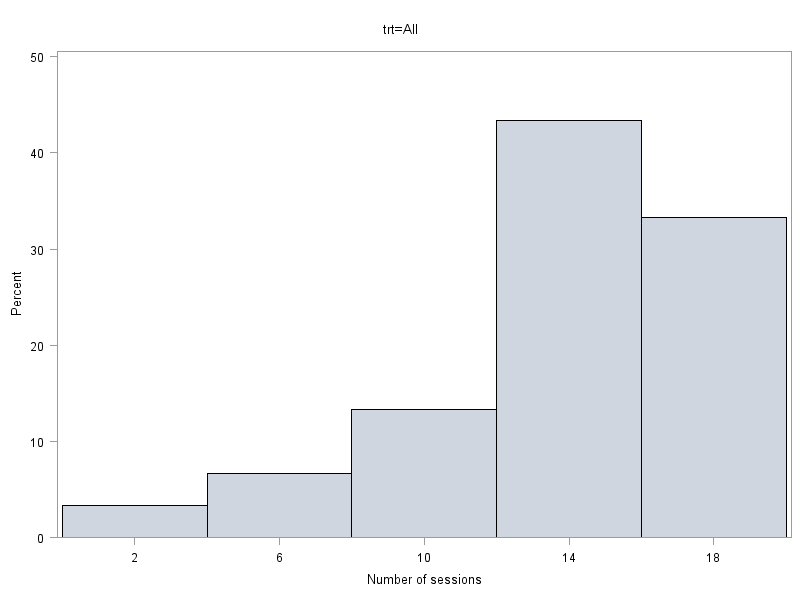


**Figure B: Histogram showing the distribution of the number of therapy sessions received by participants randomised to Group A (G-CSF and Physiotherapy, N=17)**


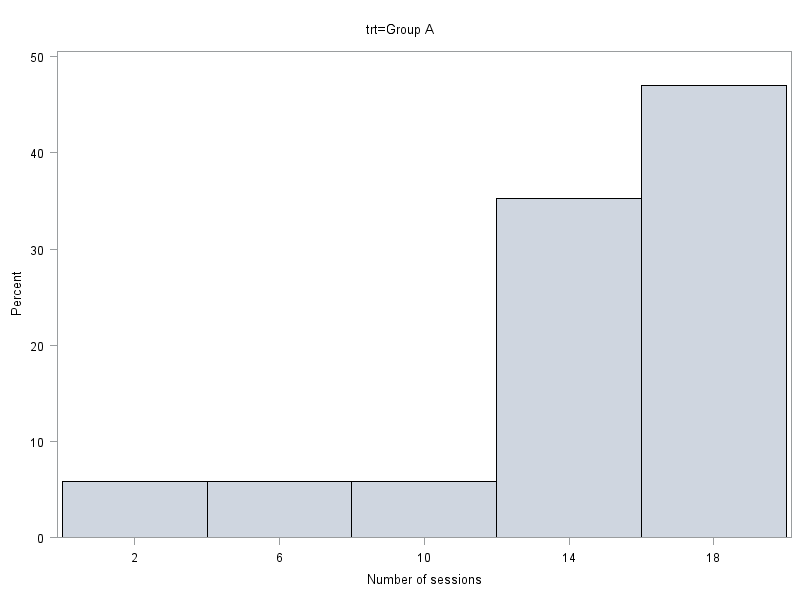


**Figure C: Histogram showing the distribution of the number of therapy sessions received by participants randomised to Group C (No G-CSF and Physiotherapy, N=13)**


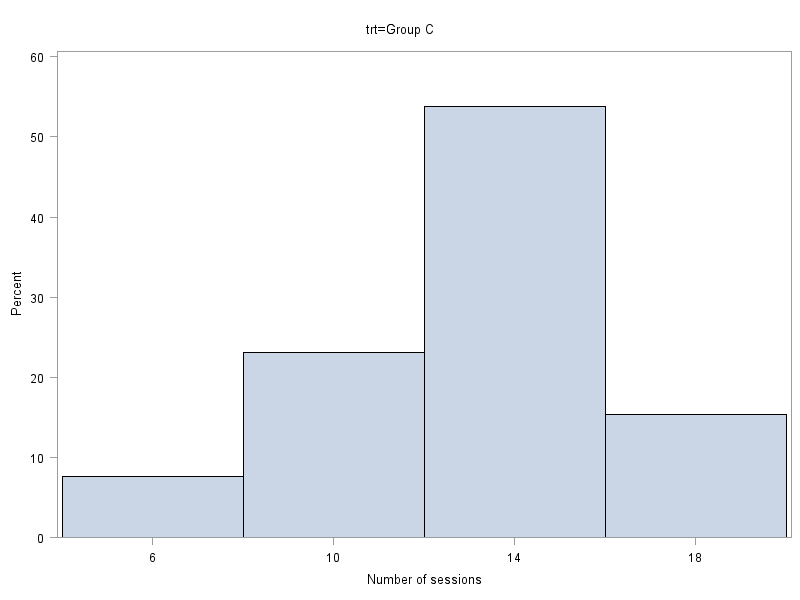

Supplement: S2 File — (A) Distribution of the number of therapy sessions received by participants randomised to receive physiotherapy. (B) Distribution of the number of therapy sessions received by participants randomised to G-CSF. (C) Distribution of the number of therapy sessions received by participants randomised to No G-CSF. (DOCX) [file pone.0161359.s003.docx]
