# Supplementary material for: Granulocyte Colony Stimulating Factor and Physiotherapy after Stroke: Results of a Feasibility Randomised Controlled Trial: Stem Cell Trial of Recovery EnhanceMent after Stroke-3 (STEMS-3 ISRCTN16714730)
Source: PLoS One. 2016 Sep 9;11(9):e0161359. doi: 10.1371/journal.pone.0161359 (PMC5017715; doi:10.1371/journal.pone.0161359)
Supplement: S1 Protocol — Protocol Version 1.3. 28th August 2012. (DOC) [file pone.0161359.s004.doc]

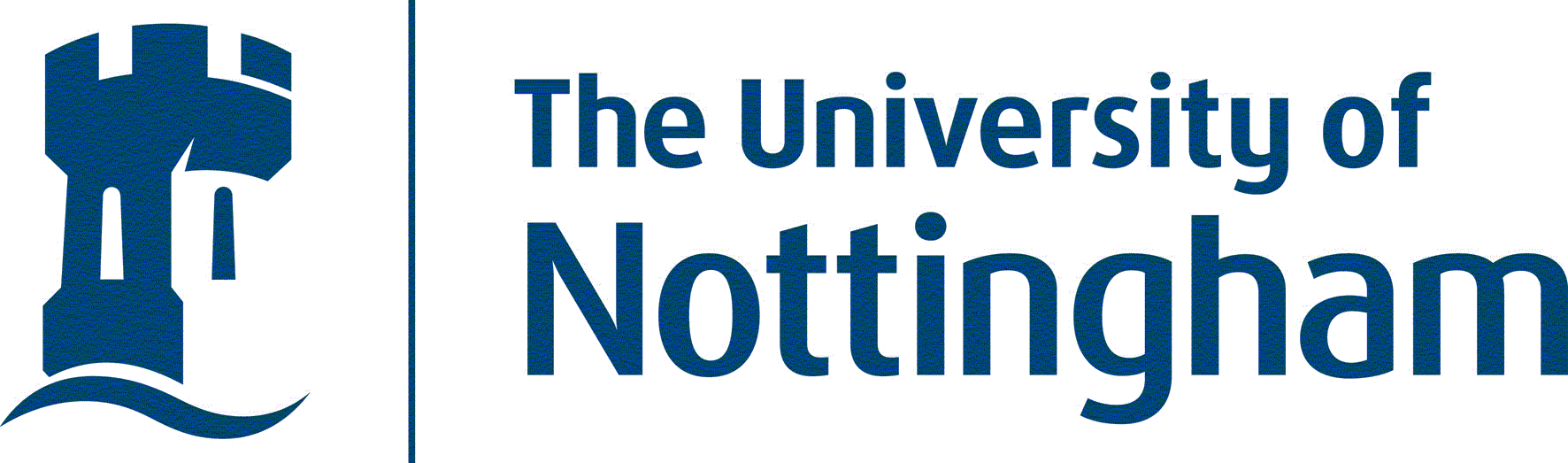


“Stem cell Trial of recovery EnhanceMent after Stroke 3’ (STEMS 3)- a pilot randomised controlled trial of G-CSF and therapy in chronic stroke

**Version 1.3**

28th August 2012

**Short title:** “Stem cell Trial of recovery EnhanceMent after Stroke 3” (STEMS 3)

**Acronym:** STEMS3

**EudraCT number:** 2011-001684-50

**Trial Registration:** [www.clinicaltrials.gov](http://www.clinicaltrials.gov/) reference

**ISRCTN:** **16714730**

**CTA reference:** 2011-001684-50 13^th^ May 2011

**NRES reference:** 11/YH/0138

**Trial Sponsor:** University of Nottingham (Ref. 11015)

**Funding Source:** PB-PG-0909-19113 - Research for Patient Benefit

TRIAL / STUDY PERSONNEL AND CONTACT DETAILS

**Sponsor:** University of Nottingham

Contact name Mr Paul Cartledge

Head of Research Grants and Contracts

Research Innovation Services

King’s Meadow Campus

Lenton Lane

Nottingham

NG7 2NR

**Chief investigator:** Dr Nikola Sprigg

(Medical expert) Clinical Associate Professor,

University of Nottingham

Phone: 0115 8231778

Fax: 0115 8231767

Email nikola.sprigg@nottingham.ac.uk

**Co-investigators:** Prof. Philip Bath

Division of Stroke

University of Nottingham

Email [Philip.bath@nottingham.ac.uk](mailto:Philip.bath@nottingham.ac.uk)

Prof. Marion Walker

Division of Rehabilitation and Ageing

University of Nottingham

Email [Marion.walker@nottingham.ac.uk](mailto:Marion.walker@nottingham.ac.uk)

Dr. Louise Connell

Research Fellow

School of Health

University of Central Lancashire

Email LAConnell@uclan.ac.uk

Rebecca Allen

Research Therapist

Division of Stroke

University of Nottingham

Email [r.allen@nottingham.ac.uk](mailto:r.allen@nottingham.ac.uk)

Dr Claire Diver

School of Nursing, Midwifery and Physiotherapy

University of Nottingham

Email Claire.Diver@nottingham.ac.uk

**Trial / Study Statistician:** Cyrille Correia

Medical Statistician

Division of Stroke

University of Nottingham

Phone: 0115 8231770

Fax: 0115 8231771

Email Cyrille.correia@nottingham.ac.uk

**Trial Pharmacist:** Sheila Hodgson

Clinical Trial Pharmacist

City Hospital

Nottingham University Hospitals NHS Trust

Phone: 01159691169 ext 57698

Fax: 01158402668

Email Sheila.Hodgson@nuh.nhs.uk

**Trial / Study Coordinating Centre:** Division of Stroke

University of Nottingham

Hucknall Road

Nottingham

NG5 1PB

**Project / Trial Manager:** No trial manager other than PI

Phone:

Fax:

Email

# SYNOPSIS

| Title | **Stem cell Trial of recovery EnhanceMent after Stroke 3 (STEMS 3)- a pilot randomised controlled trial of G-CSF and therapy in chronic stroke** |
| --- | --- |
| Acronym | STEMS3 |
| Short title | **Stem cell Trial of recovery EnhanceMent after Stroke 3** |
| Chief Investigator | Dr Nikola Sprigg |
| Objectives | Primary: To test the feasibility and tolerability of delivering G-CSF and or rehabilitation therapy in chronic stroke  Secondary: To study potential interactions between G-CSF and therapy in chronic stroke patients  To finalise the design including sample size calculation and outcome measures for the definitive trial.  These objectives will be further addressed by the qualitative sub-study which will explore the feasibility and acceptability of the recruitment process and experience of trial involvement. |
| Trial Configuration | To perform a pilot double-blind, two by two factorial design, randomised placebo-controlled trial of G-CSF and/or a 6 week course of therapy in 60 participants with previous stroke.  Semi-structured interviews in approximately 25 participants selectied puposively, non-participants, carers and clinicians. |
| Setting | Secondary care. All participants will be living at home. The intervention will be delivered at the participant’s home residence. |
| Sample size estimate | This is a feasibility study so no formal sample size calculation has been performed. The factorial design will allow us to separately assess the effect of G-CSF and therapy in chronic stroke and give additional information over two separate trials. |
| Number of participants | 60 participants |
| Eligibility criteria | Inclusion – Stroke **(90 days – 2 years after onset)** with residual motor impairment (arm or leg) with disability (modified Rankin Score >1) no longer receiving on going rehabilitation therapy |
| Description of interventions | G-CSF vs. Placebo  Subcutaneous G-CSF (Filgastrim, 1x110^6^ iu/kg) versus saline started at least 90 days after stroke onset and given daily for 5 days  Rehabilitation therapy vs. control  After receiving treatment with G-CSF or placebo participants will commence a therapy intervention, 30-45 minute sessions three times a week, for six weeks. Participants randomised to control will receive no therapy intervention. |
| Duration of study | Overall 24mths - planned start date 01.08.2011 finish date 31.07.2013  Each participant will be followed up for 365 days after randomisation |
| Randomisation and blinding | Randomisation will be 1:1 G-CSF: placebo and therapy:control and performed by computer generated sequence.  The IMP (G-CSF or saline placebo) will be dispensed in identical syringes appropriately labelled as trial medication so the researchers and participant will remain blinded to treatment allocation.  Outcome assessors will be blind to treatment allocation. |
| Outcome measures | Feasibility: proportion of participants receiving all 5 G-CSF/placebo injections; proportion of participants receiving all therapy sessions.  Acceptability- proportion of participants screened who are eligible for enrollment who give consent;  Tolerability- Adverse events (headache, backache) reported after G-CSF administration; proportion of participants who withdraw or decline rehabiliation therapy sessions.  Secondary outcome measures-  Haematological (FBC, WCC, CD34, PLT)  Post therapy intervention (day 45, day 90, and end of follow-up day 365): Motor function (RMA); change in dependency (modified Rankin Scale shift); change in disability (change in BI); quality of life (EuroQoL); care giver burden;Change in balance (Berg Balance Scale (BBS); Change in mental state (Mini-Mental State Examination (MMSE); Change in mood (Zung Depression Scale); Change in dependency (Nottingham Extended Activities of Daily Living (NE-ADL).    Safety, end of follow-up (day 365): Death (cause); recurrence; infection; SAE’s.  Effect of the intervention on carer burden will be assessed incase where the carer gives consent to complete a questionairre. |
| Statistical methods | As this is a pilot study the analysis will be mainly descriptive. The primary and secondary measures will be compared between participants randomised to G-CSF versus placebo (intention-to-treat), therapy intervention versus control (intention to treat). No subgroup analyses will be performed |
| Qualitative sub-study | Approximately 25 participants  Semi structured interviews to take place during the duration of the main trial |

# ABBREVIATIONS

| ADR | Adverse Drug Reaction |
| --- | --- |
| AE | Adverse Event |
| CD34 | Colony dependent 34 count |
| CF | Informed Consent Form |
| CI | Chief Investigator overall |
|  |  |
| CRF | Case Report Form |
|  |  |
| DAP | Data Analysis Plan |
| DMC | Data Monitoring Committee |
|  |  |
| EMEA | European Agency for the Evaluation of Medicinal Products |
| EOT | End of Trial |
| FBC | Full blood count |
| GCP | Good Clinical Practice |
| G-CSF | Granulocyte colony stimulating factor |
| IMP | Investigational Medicinal Product |
|  |  |
| MHRA | Medicines and Healthcare products Regulatory Agency |
| NHS | National Health Service |
| NUH | Nottingham University Hospitals |
| OT | Occupational therapy |
| P/GIS | Parent / Guardian Information Sheet |
| PI | Principal Investigator at a local centre |
| PIS | Participant Information Sheet |
| PLT | Platelet studies |
| PT | Physiotherapy |
| REC | Research Ethics Committee |
| R&D | Research and Development department |
|  |  |
| SAE | Serious Adverse Event |
| SAR | Serious Adverse Reaction |
| SmPC | Summary of Product Characteristics |
| SUSAR | Suspected Unexpected Serious Adverse Reaction |
|  |  |
| TMG | Trial Management Group |
| TSC | Trial Steering Committee |
| WCC | White cell count |

**TABLE OF CONTENTS**

SYNOPSIS [4](#__RefHeading___Toc163818165)

ABBREVIATIONS [6](#__RefHeading___Toc163818166)

TRIAL / STUDY BACKGROUND INFORMATION AND RATIONALE [10](#__RefHeading___Toc163818167)

DETAILS OF INVESTIGATIONAL MEDICINAL PRODUCT(S) [11](#__RefHeading___Toc163818168)

Description [11](#__RefHeading___Toc163818169)

Product Characteristics [11](#__RefHeading___Toc163818170)

Description 12

Manufacture, packaging and labelling [12](#__RefHeading___Toc163818172)

Storage, dispensing and return [12](#__RefHeading___Toc163818173)

Placebo [12](#__RefHeading___Toc163818174)

Known Side Effects 13

TRIAL / STUDY OBJECTIVES AND PURPOSE 13

PURPOSE 13

PRIMARY OBJECTIVE 13

SECONDARY OBJECTIVES [13](#__RefHeading___Toc163818179)

TRIAL / STUDY DESIGN 14

TRIAL / STUDY CONFIGURATION 14

Primary endpoint 15

Secondary endpoint 15

Safety endpoints 16

Stopping rules and discontinuation 16

RANDOMIZATION AND BLINDING 16

Maintenance of randomisation codes and procedures for breaking code 17

TRIAL MANAGEMENT 17

Data Safety Monitoring Committee (DSMC): 17

DURATION OF THE TRIAL / STUDY AND PARTICIPANT INVOLVEMENT 17

End of the Trial 17

SELECTION AND WITHDRAWAL OF PARTICIPANTS 18

Recruitment 18

Inclusion criteria for main study 21

Exclusion criteria for main study 21

Inclusion criteria for carer sub-study 22

Exclusion criteria for carer sub-study 22

Expected duration of participant participation 22

Removal of participants from therapy or assessments 22

Informed consent 22

TRIAL / STUDY TREATMENT AND REGIMEN 23

Blood Samples 24

Compliance 24

Accountability for drugs & placebos 24

Management of study drug overdose 24

Criteria for terminating trial 24

STATISTICS 25

Methods 25

Sample size and justification 25

Assessment of efficacy 25

Assessment of safety 25

Procedures for missing, unused and spurious data 25

Definition of populations analysed 26

ADVERSE EVENTS 26

Definitions 26

Causality 27

Reporting of adverse events 27

SUSARs 28

Trial Treatment Related SAEs 28

Participant removal from the study due to adverse events 29

QUALITATIVE SUB STUDY 29

Aim 29

Objectives 29

Design 29

Participants 29

Sampling 30

Data collection 30

Topic guide 30

Data analysis 31

ETHICAL AND REGULATORY ASPECTS 31

ETHICS COMMITTEE AND REGULATORY APPROVALS 31

INFORMED CONSENT AND PARTICIPANT INFORMATION 32

RECORDS 32

Drug accountability 32

Case Report Forms 33

Source documents 33

Direct access to source data / documents 33

DATA PROTECTION 33

QUALITY ASSURANCE & AUDIT 34

INSURANCE AND INDEMNITY 34

TRIAL CONDUCT 34

TRIAL DATA 34

RECORD RETENTION AND ARCHIVING 35

DISCONTINUATION OF THE TRIAL BY THE SPONSOR 35

STATEMENT OF CONFIDENTIALITY 35

PUBLICATION AND DISSEMINATION POLICY 35

USER AND PUBLIC INVOLVEMENT 36

STUDY FINANCES 36

Funding source 36

Participant stipends and payments 36

SIGNATURE PAGES 37

REFERENCES 38

# TRIAL / STUDY BACKGROUND INFORMATION AND RATIONALE

Stroke is the major cause of disability in the UK, 130,000 people a year having a stroke in England and Wales, with half of survivors being dependent on others six months later. Despite this the majority of patients are no longer receive ongoing rehabilitation therapy beyond three to six months after stroke. With stroke increasing almost exponentially with age, plus the changes in age demographics of the UK population, the number of people living in the community after having a stroke is set to rise over the forthcoming decades. The cost of stroke to the NHS is estimated to be over £2.5 billion per year.

Neuroplasticity:
Modern concepts of recovery after stroke include neuroplasticity, the brains ability to undergo dynamic change. Rehabilitation promotes functional recovery but it is not known if this recovery is optimal. The magnitude could be further enhanced by use of drug treatments.

Recovery of function after stroke occurs to a variable degree through a number of restorative pathways, the brain having a plastic potential for remodelling and repair. There is now evidence that plasticity extends beyond the sub-acute stage of stroke into more chronic stages of stroke.^1^

There is increasing interest in the use of interventions which may enhance these normal restorative events after stroke,^2^ including through the use of stem cells (cells capable of self renewal and differentiating into multiple cell types). One source of stem cells is to mobilise the release of endogenous haematopoietic (CD34+) bone marrow stem cells into the circulation, e.g. with granulocyte-colony stimulating factor (G-CSF). G-CSF is routinely used, and licensed, for mobilising stem cells for transplantation in haematological malignancy.^3^

G-CSF in experimental stroke:

G-CSF mobilises bone marrow stem cells into the circulation and improves motor recovery in experimental models of ischaemic stroke ^4,5,6,7^ with reduced infarct size, neurological impairment, and improved function.^8^ Likely mechanisms include reducing apoptosis (thus rescuing cells that could contribute to recovery); enhancing neurogenesis, through local brain progenitors and/or by mobilising peripheral blood stem cells (PBSCs), and angiogenesis/vascularisation. G-CSF also enhanced recovery after experimental intracerebral haemorrhage.^9^

G-CSF for treating clinical stroke:

G-CSF is currently being tested in clinical stroke. A few small RCT have examined GCSF in clinical stroke,^10,11,12^ and in meta-analysis G-CSF was associated with a non-significant reduction in combined death and dependency in two small trials (n = 46 participants) although there was substantial heterogeneity in this result.^13^ The trials were too small to adequately study the effects of G-CSF on functional outcome, although G-CSF did increase peripheral blood stem cells in stroke patients. Larger explanatory phase II clinical trials are now on-going in acute and subacute stroke. ^14,15^

G-CSF in chronic stroke:

In chronic experimental stroke models, rats given G-CSF and SCF had improved outcome^16^  while mice given G-CSF or SCF in chronic stroke promoted mobilisation of PBSCs, with increased entry into the brain and angiogenesis.^17^

As yet no clinical trial has tested G-CSF in chronic stroke. Our proposed trial will address this approach in assessing whether G-CSF can enhance recovery in patients with chronic disability after stroke.

Rehabilitation therapy in chronic stroke

Unfortunately the majority of patients are no longer receive ongoing rehabilitation therapy beyond three to six months after stroke. Therapy in stable stroke can promote recovery possibly via stimulating neuroplasticity^18,19^ The dose, method and intensity of therapy appears to be important^20^ , with exercise therapy most effective when delivered as high intensity task-specific practise.^21^ A number of studies have assessed therapy in stroke patients living at home, and while there seems to be benefits in receiving therapy at home^22^ there is not enough data to say whether therapy in chronic stroke is effective long term.^23^  However we do know that routine therapy is essentially finished at 6 months post stroke, with most finishing at 6 weeks after community stroke team (CST) and early supported discharge services (ESD) have withdrawn. In some cases CST continues but this is usually complete by 6 months. The mainstay of intervention at this stage is towards secondary prevention. This makes this area a naturally occurring control arm as there is unlikely to be spontaneous recovery at this stage without intervention.

Therapy can be delivered in the community, as is the case with ESD teams and CST. While patients and carers report a desire for continued therapy, there is little data documenting whether continued treatment in the chronic stages of stroke is feasible and or well tolerated.

G-CSF and rehabilitation therapy in chronic stroke

If G-CSF is effective at improving recovery in chronic stroke it is likely that this recovery can be optimised by the combination of G-CSF and rehabilitation therapy. This is seen in experimental models of stroke where improved functional outcome is demonstrated in models containing stimulation, repetition and practice.^24^

If the intervention is effective it is possible it may reduce carer burden as participants will become more independent. Conversely the study may cause additional carer burden – arranging study visits, time taken for therapy – so we will seek consent from the participants carer to measure carer burden with a short questionnaire. If there is no carer or the carer does not consent to this we will not collect this data for that participant.

Most acute stroke trails follow up participants to 90 days whereas follow up to 365 days in more in keeping with rehabilitation trials in chronic stroke. This is consistent with comments given by stroke consumer groups who recommended follow up to 365 days. We will follow up at day 45 and 90, which is consistent with similar trials of G-CSF, and day 365 which is consistent with chronic stroke rehabilitation trials.

## DETAILS OF INVESTIGATIONAL MEDICINAL PRODUCT(S)

### Description

Subcutaneous human recombinant G-CSF (Filgrastim 1x10^6^ units/kg equivalent 10µg/kg) versus placebo (Sodium Chloride 0.9% 0.03ml/kg) will be given for 5 days.

## Product Characteristics

Filgrastim injection 300μg/ml (Neupogen, Amgen) is a licensed product (PL 16216/0038) and a summary of the product characteristics is available for investigators (see appendix). Filgrastim is a clear, colourless solution for injection.

Sodium Chloride 0.9% Injection (Baxter Healthcare) is a licensed product and a summary of product characteristics is available for investigators (see appendix) It is a clear, colourless solution for injection

### Description

Standard NHS supplies will be used. Subject specific treatment packs will be prepared and dispensed by pharmacy. The individual treatment packs will contain a total of five doses and will be packaged and labelled in accordance with [Annex 13 of Volume 4 of The Rules Governing Medicinal Products in the EU: Good Manufacturing Practices](http://ec.europa.eu/enterprise/pharmaceuticals/eudralex/vol-4/pdfs-en/an13final_24-02-05.pdf). The syringes are labelled only as trial medication so the researchers and participant will remain blinded to treatment allocation.

### Manufacture, packaging and labelling

For each subject, the required volume of Filgrastim injection 300μg/ml, to provide a dose of 10μg/kg will be calculated. Under aseptic conditions, the dose will be drawn into a 3ml Becton Dickinson luer lock tuberculin syringe and sealed with a Baxa luer lock syringe cap. The maximum volume for a single injection will be 2ml. For subjects requiring a dose volume of more than 2mls, the total volume required will be divided into 2 injections of approximately equal size. Prefilled syringes will be stored between 2 and 8◦C and assigned a 7 day expiry (Ref Handbook on Injectable Drugs, Lawrence A Trissel, 11^th^ edition)

Sufficient syringes to provide a 5 day course of treatment will be packed into a suitable container and labelled in accordance with [Annex 13 of Volume 4 of The Rules Governing Medicinal Products in the EU: Good Manufacturing Practices](http://ec.europa.eu/enterprise/pharmaceuticals/eudralex/vol-4/pdfs-en/an13final_24-02-05.pdf). (see sample label provided)

.

### Storage, dispensing and return

Following randomisation, the IMP will be prescribed, prepared and dispensed on an individual participant basis. A prescription will be written with details of the treatment pack number required. Each treatment pack will be a numbered container with a 5 day supply of either G-CSF or placebo, in accordance with the randomisation schedule. This will be dispensed by NUH clinical trials pharmacy staff who are unblinded to the treatment allocation.

Once the first dose has been administered the IMP will be stored at 2-8◦C in the participants refrigerator for daily adminstration by the research nurse at the participants home for 5 consecutive days.

The pharmacist will maintain records of the distribution of IMP the research nurse will check the treatment pack number is correct for the subject and record administration of the IMP . The research nurse will return any unused IMP to NUH Trials pharmacy.

### Placebo

For each subject, the required volume of Sodium Chloride 0.9% Injection, to provide a dose of 0.03ml/kg will be calculated. Under aseptic conditions, the dose will be drawn into a 3ml Becton Dickinson luer lock tuberculin syringe and sealed with a Baxa luer lock syringe cap. The maximum volume for a single injection will be 2ml. For subjects requiring a dose volume of more than 2mls, the total volume required will be divided into 2 injections of approximately equal size. Prefilled syringes will be stored between 2 and 8◦C and assigned a 7 day expiry (Ref Information on file at MHRA Site Number 3457, MIA(IMP)19162)

Sufficient syringes to provide a 5 day course of treatment will be packed into a suitable container and labelled in accordance with [Annex 13 of Volume 4 of The Rules Governing Medicinal Products in the EU: Good Manufacturing Practices](http://ec.europa.eu/enterprise/pharmaceuticals/eudralex/vol-4/pdfs-en/an13final_24-02-05.pdf) (see sample label provided)

### Known Side Effects

The most commonly reported undesirable effect is mild to moderate transient musculoskeletal pain. Leukocytosis (WBC> 50 x 10^9^/L) and transient thrombocytopenia (platelets < 100 x 10^9^/L) are well recognized effects. Transient, minor increases in alkaline phosphatase, lactate dehydrogenase, aspartate aminotransferase and uric acid have been reported but these were without clinical sequelae. Exacerbation of arthritic symptoms has been observed very rarely. Headaches, believed to be caused by filgrastim, have been reported. Asymptomatic cases of splenomegaly and very rarely cases of splenic rupture have been reported following administration of granulocyte-colony stimulating factors (GCSFs).

No side effects reported for subcutaneous injection of placebo (saline).

# TRIAL / STUDY OBJECTIVES AND PURPOSE

## PURPOSE

Ultimately we plan to run a definitive factorial trial to test the hypotheses that

1. G-CSF treatment in participants with previous stroke will promote recovery determined as improved motor function.

2. Rehabilitation therapy intervention (PT and/or OT) in participants with previous stroke will promote recovery determined as improved motor function.

3. There may be additive benefit of giving G-CSF followed by rehabilitation therapy in participants with previous stroke.

In the present project STEMS3 we wish to collect data to finalise the design of such a trial.

## PRIMARY OBJECTIVE

Assess the safety, feasibility of administration, and tolerability of delivering G-CSF and/or therapy (PT and/or OT) in the community in chronic stroke patients.

## SECONDARY OBJECTIVES

1. Study potential interaction between G-CSF and therapy (PT and/or OT) in chronic stroke patients. Whilst experimental data suggests that neuroplasticity and recovery is enhanced by combination of drug and therapy stimulation this will be further assessed by the factorial design of this study.

2. Finalise the design including the sample size calculations and outcome measures for the definitive trial, by obtaining information on functional measures, the spectrum of disability among trial recruits and the completeness of outcome data.

# TRIAL / STUDY DESIGN

See study flow chart page 19

## TRIAL / STUDY CONFIGURATION

Blinded outcome, single centre, two by two factorial design, randomised (1:1) placebo-controlled trial of G-CSF (subcutaneous injection daily for 5 days) and/ or a 6 week course of therapy (PT and/or OT) in 60 participants with previous stroke.

**Therapy + Therapy** -

**G-CSF +** (n=15) (n=15) *Total n=30*

**G-CSF -** (n=15) (n=15) *Total n=30*

*Total n=30 Total n=30*

Assessments:

Clinical assessment; Baseline (Day 0), end of G-CSF treatment (day 5), end of therapy treatment (day 45), 3 month follow up (day 90) and 1 year follow up (day 365).

Haematological – FBC (CD34, WCC, PLT) Day 5 (end of G-CSF treatment)

### Primary endpoint

Feasibility: proportion of participants receiving all 5 G-CSF/placebo injections; proportion of participants receiving all therapy sessions.

Acceptability- proportion of participants screened who are eligible for enrollment who give consent;

Tolerability- Adverse events (headache, backache) reported after G-CSF adminisitration; proportion of participants who withdraw or decline rehabiliation therapy sessions.

### Secondary endpoint

Haematological - Post treatment (day 5): CD34+ count; full blood count, platelet studies. Blood (approximately 10-15mls) will be taken at day 0 (randomisation), and day 5 by the investigator or research nurse. The effect of G-CSF on CD34+, WCC, and platelet count will be determined in all participants. FBC and WCC will be analysed by the haematology laboratory at NUH, CD34 and PLT or by the University research laboratory at NUH.

Post therapy intervention (day 45) Motor function (RMA); change in dependency (modified Rankin Scale shift); change in disability (change in BI); quality of life (EuroQoL).

Three month follow-up (day 90): Motor function (RMA); change in dependency (modified Rankin Scale shift); change in disability (change in BI); quality of life (EuroQoL).

Change in balance (Berg Balance Scale (BBS). Change in mental state (Mini-Mental State Examination (MMSE). Change in mood (Zung Depression Scale). Change in dependency (Nottingham Extended Activities of Daily Living (NE-ADL).

**End follow up (day 365)**: Motor function (RMA); change in dependency (modified Rankin Scale shift); change in disability (change in BI); quality of life (EuroQoL).

**Change in balance (Berg Balance Scale (BBS). Change in mental state (Mini-Mental State Examination (MMSE). Change in mood (Zung Depression Scale). Change in dependency (Nottingham Extended Activities of Daily Living (NE-ADL).**

Participants carers will be asked to give consent to complete a care giver burden questionaire at day 45, day 90 and day 365 to assess if the intervention has any effect on care giver burden.

Participants will be asked to give consent to participate in the qualitative substudy.

Participants who have not been consented to the day 365 follow up at entry to the trial will be reconsented at day 45, day 90 or contacted to ask if they would like to take part in the extended follow up and qualitative substudy.

Fidelity of treatment will be assessed to ensure pateints get the correct intervention.

Intervention records will be collected and checked.

### Safety endpoints

Safety, end of follow-up (day 90): Death (cause); recurrence; infection; SAE’s.

### Stopping rules and discontinuation

Participants may withdraw consent at any time. Study medication maybe stopped at any time by the investigator or treating physician if deemed advisable. For un-blinding procedure see below.

## RANDOMIZATION AND BLINDING

Randomisation will be 1:1 G-CSF: placebo and therapy:control, and involve computerised minimisation on key prognostic factors: age; gender; time from stroke; motor function (Rivermead Motor Assesment, RMA). Randomisation will be performed by the STU.

All participants eligible for inclusion and for whom consent has been obtained will be randomised centrally using a secure internet site in real-time. Randomisation will be performed using:

1. Minimisation on key prognostic/logistical baseline factors:
   1. Age (<70/>70 yrs)
   2. Sex (male/female)
   3. Time from stroke

d. Disability measure (Rivermead)

This approach ensures concealment of allocation, minimises differences in key baseline variables, and slightly improves statistical power.

In the event that the website cannot be accessed, participants may be randomised by telephoning one of a series of emergency telephone numbers. These participants will be randomised without stratification or minimisation.

Randomisation will allocate a number corresponding to a treatment pack and the participant will receive treatment from the allocated numbered pack.

**Blinding:**

The drug will be prescibed and dispensed on an individual participant basis, in 5 day treatment packs. The drug (G-CSF or saline placebo) will be dispensed in identical syringes appropriately labelled as trial medication so the researchers and participant will remain blinded to treatment allocation.

Participants randomised to control therapy will receive no therapy visits so will not be blinded. The outcome assessors (research nurse) will be blinded to treatment allocation. Study statistician and the data monitoring committee (who are un-blinded) will not have any contact with study participants or outcome assessors.

### Maintenance of randomisation codes and procedures for breaking code

Un-blinding of a participant can be performed at any time by contacting the co- investigator Professor Philip Bath (emergency phone contact) or pharmacy in the event of a medical emergency when treatment is dependent on knowledge of the actual drug received.

In the even of breaking the treatment code this will normally be recorded as part of managing an SAE (see below for more details). In cases where un-blinding was not associated with an SAE, such actions will be reported in a timely manner (notification of Sponsor immediately as practicable by phone or fax, followed by a written narrative of the event within 48 hours).

*.*

## TRIAL MANAGEMENT

The project will be managed through the Stroke Trials unit. The steering committee will comprise all grant holders. It will monitor all aspects of the study and progress to insure protocol adherence and quality of trial data. It will also facilitate the dissemination of results and the contact between the team and the stakeholders (e.g. Clinicians, Consumer Group, Stroke Association etc). The steering committee will meet formally at least four times, depending on the stage of trial. However it is anticipated that much of the routine work can be done via email. Day to day managment will be responsibility of the trial nurse supervised by NS. NS willl monitor progress in respect of project milestones and provide apropriate reports to the steering committee. Financial management will be the responsibility of NS.

## Data Safety Monitoring Committee (DSMC):

The DSMC will review un-blinded safety data (without formal statistical analysis) on a regular basis, after 10 participants have been recruited and followed for 45 days. The frequency of DSMC review can be increased if appropriate. DSMC will advise the Steering Committee on any safety issues; any decision to stop the study prematurely will be based on asymmetric stopping rules^25^. The committee comprises: Professor Martin Dennis (chair of DSMC in both previous STEMS trials and experienced trialist), Dr Ashit Shetty (Stroke Physician, Nottingham). Data for the DSMC will be prepared by (Trial Statistician, Nottingham).

## DURATION OF THE TRIAL / STUDY AND PARTICIPANT INVOLVEMENT

The participant’s involvement in the trial will last 90 days, from consent and randomisation (day 0) until final follow up at 365 days. Treatment period will be for 1 day.

Enrolment will begin when the study has obtained full regulatory approval and cease when the final (60^th^) participant has completed follow up period.

### End of the Trial

The trial will end when the final participant has completed treatment period and follow up (Day 365).

## SELECTION AND WITHDRAWAL OF PARTICIPANTS

### Recruitment

Participants will be recruited from Nottingham University Hospitals stroke services, NHS Nottingham City and NHS Nottinghamshire County community stroke teams. The initial approach will be from a member of the participant’s usual care team (which may include the investigator and/or research nurses and/or therapist). The investigator or their nominee, e.g. from the usual care team (including research team), will inform the participant about the trial and a participant information sheet will be provided. Patient and GP contact details will be collected. Informed consent will be taken from participants at this point of contact to perform a telephone assessment of function (modified Rankin scale) at 3 months after the stroke.

On the basis of the telephone assessments, if the participant is eligible and interested, a participant information sheet will be posted to the participant. The participant’s GP will be informed about the study. Should the GP have concerns about their patient participating in the study, they will be asked to contact the local hospital research centre. It is important to note that GPs will not be involved in screening and recruiting patients.

Participants will be contacted a week later to assess their views about participation in the trial and to answer any questions. All participants will be booked to come to NUH City Campus research centre for further discussion, and if agreeable, enrolment and randomisation into the study.

If needed, the usual interpreter and translator services will be available to assist with discussion of the trial, the participant information sheets, and consent forms, but the consent forms and information sheets will not be available printed in other languages. It will be explained to the potential participant that that entry into the trial is entirely voluntary and that their treatment and care will not be affected by their decision. It will also be explained that they can withdraw at any time but attempts will be made to avoid this occurrence. In the event of their withdrawal it will be explained that their data collected so far cannot be erased and we will seek consent to use the data in the final analyses where appropriate.

Participants who have carers will be asked if their carers wish to complete a carer burden questionnaire. The carer will be issued with an information sheet and if they wish to take part, full informed consent will be taken. This carer burden questionnaire will involve a short interview and questionnaire which will last approximately 20 minutes and will be performed three times, at baseline, day 45 and day 90.

Participants in STEMS3, potential participants for STEMS3 and clinicians involved in identifying participants for STEMS3 will be asked if they wish to be involved in the qualitative sub study. Potential participants will be issued with an information sheet and if they wish to take part, full informed consent will be taken. Participants will complete a semi-structured interview, which will last approximately 1 hour.

For the qualitative sub study, participants will be selected purposively from those who have consented to inclusion in the trial.


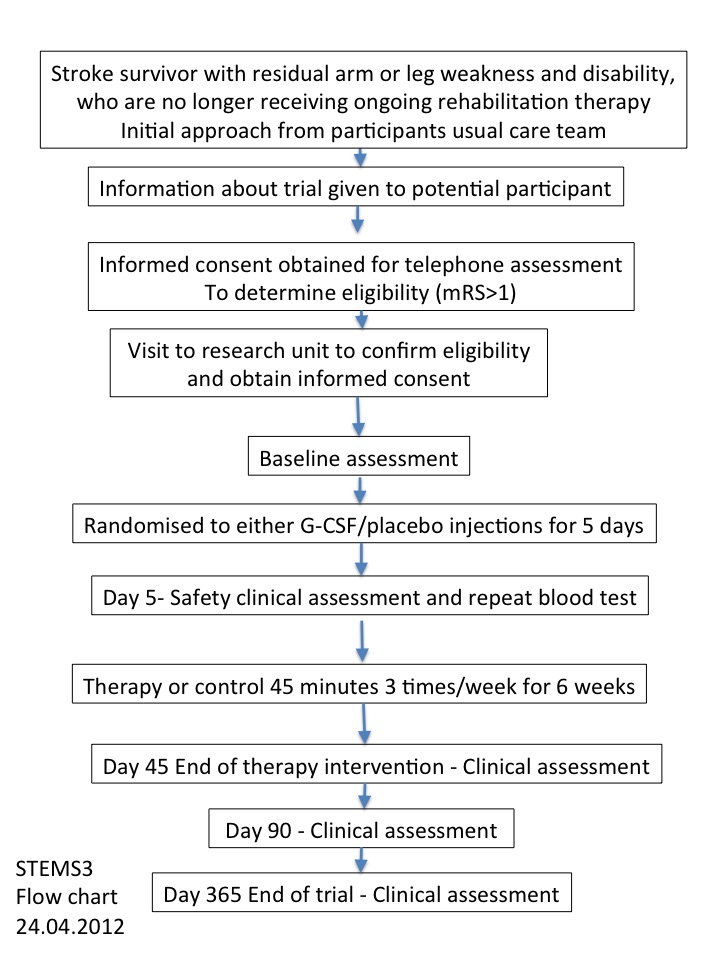


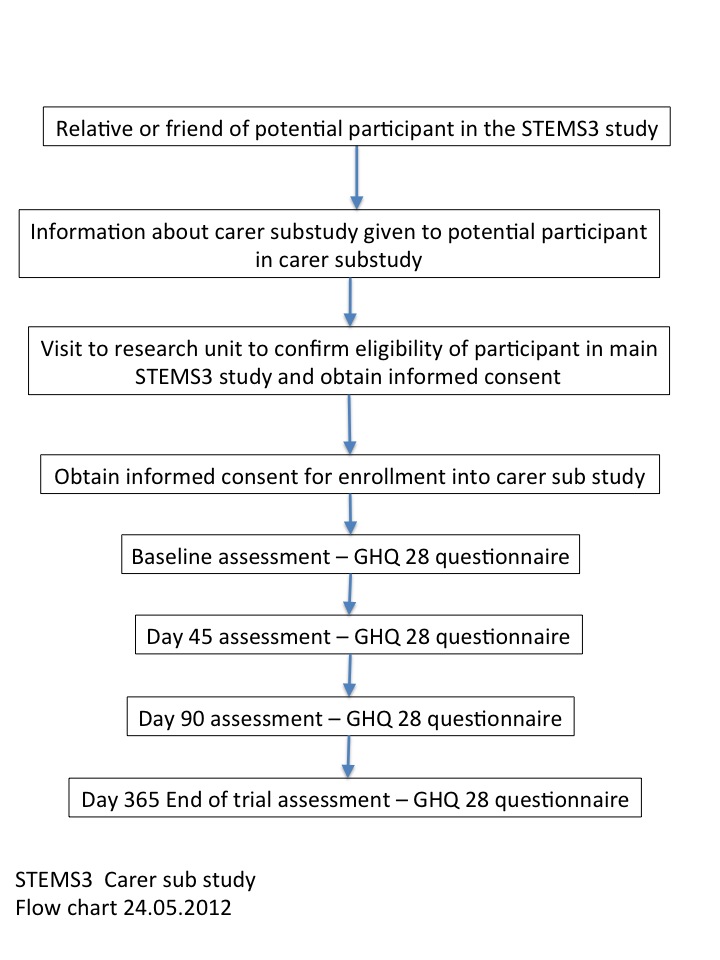


###
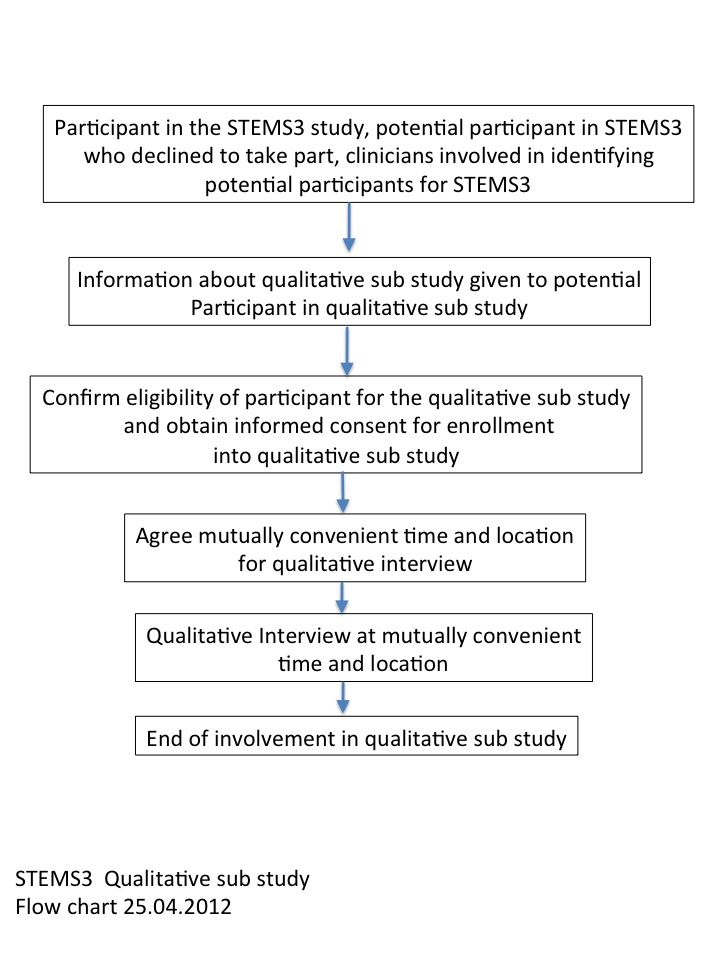


### Inclusion criteria for main study

Adults (18 years and over) with motor impairment (arm or leg) with residual disability (modified Rankin Score >1) due to stroke **(90 days – 2 years post onset)** no longer receiving on going rehabilitation therapy.

### Exclusion criteria for main study

Lack of residual motor deficit; significant cognitive impairment that will impede ability to complete assessments, diagnosis likely to interfere with outcome or rehabilitation (e.g. terminal illness), still receiving post stroke rehabilitation; pregnancy; other exclusions of G-CSF (as per British National Formulary –see SmPC).

### Inclusion criteria for carer sub-study

Relative or friend of participant in the main study

### Exclusion criteria for carer sub-study

Significant cognitive impairment that will impede ability to complete assessments

**Inclusion criteria for qualitative sub-study**

Participant in STEMS3, screened for inclusion in STEMS3 but declined to participate, clinicians involved in identifying participants for screening into STEMS3

**Exclusion for qualitative sub-study**

Communication difficulties or cognitive impairments which prevent participation in interviews

### Expected duration of participant participation

Study participants will be participating in the study for 365 days.

### Removal of participants from therapy or assessments

Participation in the trial is voluntary and participants are free to withdraw from the trial at any stage without giving a reason. Un-blinding of a participant can be performed at any time by contacting the co-investigator Professor Philip Bath if it is deemed necessary to determine the proper medical management of a participant. This is standard practice in other studies run from University of Nottingham Stroke Trials Unit (STU). Study medication may be stopped at any time by the investigators or any treating clinician if deemed in the participant’s best interest. Treatment (with G-CSF/placebo) will be given on top of ‘best medical care’.

Participants may be withdrawn from the trial either at their own request or at the discretion of the Investigator. The participants will be made aware that this will not affect their future care. Participants will be made aware (via the information sheet and consent form) that should they withdraw the data collected to date cannot be erased and may still be used in the final analysis.

Enrolled participants who withdraw before randomisation can be replaced (though keeping their trial ID), but participants who withdraw after randomisation will not be replaced.

### Informed consent

All participants (for the main study, carer sub study and qualitative sub-study) will provide written informed consent.

If the potential participant is not able to provide informed consent they will not be enrolled in the study. The Informed Consent Form will be signed and dated by the participant before they enter the trial. The Investigator (or nominee) will explain the details of the trial and provide a Participant Information Sheet, ensuring that the participant has sufficient time to consider participating or not. The Investigator will answer any questions that the participant has concerning study participation. Potential participants will be given as long as they need to consider whether to consent. Informed consent will be collected from each participant before they undergo any interventions related to the study. For the main study one copy of the consent form will be kept by the participant, one will be kept by the Investigator, and a third will be retained in the participant’s hospital records. . For the carer sub study one copy of the consent form will be kept by the participant and one will be kept by the Investigator.

Should there be any subsequent amendment to the final protocol, which might affect a participant’s participation in the trial, continuing consent will be obtained using an amended consent form, which will be signed by the participant.

## Participants already recruited to the trial will be approached to obtain consent to the 365 day follow up and invited to consider participation in the qualitative sub-study. Those interested in taking part in the qualitative sub-study will provide written informed consent prior to participation.

## TRIAL / STUDY TREATMENT AND REGIMEN

IMP: G-CSF (Filgrastim equivalent 10µg/kg) or placebo (saline) will be given once daily as a subcutaneous injection, for 5 consecutive days. The research nurse will visit the participant’s house daily for 5 consecutive days to administer the injection.

Treatment (with G-CSF/placebo) will be given on top of ‘best medical care’, including treatment with appropriate secondary prevention.

Therapy intervention: Starting the week of G-CSF (or placebo) treatment, participants will either recieve a therapy intervention, (30-45 minute sessions three times a week for six weeks), or control (no therapy intervention). Therapy will commence the same week as the injections to allow for the bone marrow stem cells to be mobilised into the circulation at the time the therapy stimulates any potential plasticity in the brain.

Therapy will be standard care therapy, as is delivered by stoke therapist (PT or OT) in the community stroke team. Content of therapy will vary, being dependent on the participant’s needs, as assessed by the therapists. OT will concentrate on self-care and extended ADL tasks that address participant’s goals and functional restrictions. PT will be based on participant’s needs, based on intense task specific practice and work towards measurable achievable goals.

Participants randomised to control therapy will receive no therapy visits.

Therapists will record content of therapy delivered, as has been utilised in previous rehabilitation trials.

#### Participant measures

| **Time (days)** | **Screen** | **1** | **5** | **45** | **90** | **365** |
| --- | --- | --- | --- | --- | --- | --- |
| **Inclusion** | **+** | **+** |  |  |  |  |
| **Consent** | **+** | **+** |  |  |  |  |
| **Randomise** | **+** | **+** |  |  |  |  |
| **FBC, Platelet studies, CD34+** |  | **+** | **+** |  |  |  |
| **NIHSS** |  | **+** |  | **+** | **+** | **+** |
| **mRS** |  | **+** |  | **+** | **+** | **+** |
| **SAEs** |  | **+** | **+** | **+** | **+** | **+** |
| **BI** |  | **+** |  | **+** | **+** | **+** |
| ***Rivermead*** |  | ***+*** |  | ***+*** | ***+*** | ***+*** |
| ***EuroQol*** |  |  |  | ***+*** | ***+*** | ***+*** |
| ***Berg Balance Scale*** |  | ***+*** |  | ***+*** | ***+*** | **+** |
| ***MMSE*** |  | ***+*** |  | ***+*** | ***+*** | **+** |
| ***Zung Depression Scale*** |  | ***+*** |  | ***+*** | ***+*** | **+** |
| ***NE-ADL*** |  | ***+*** |  | ***+*** | ***+*** | **+** |
| ***Qualitative sub-study***  ***Semi-structured interviews*** |  | ******* | ******* | ******* | ******* | ***** |

**** Semi-structured interview may take place at any time during inclusion in the trial***

Assessments:

Clinical assessment by face to face interview; Baseline (Day 0), end of G-CSF treatment (day 5), end of therapy intervention (day 45) and end of follow up - day 90.

Haematological – FBC (CD34, WCC), platelet studies Day 0 and day 5 (end of G-CSF treatment). The research nurse will take the blood tests at the participant’s home when they visit to administer the injection.

Researchers will not contact the participant or their family directly at Day 90, they will first contact the participant’s general practitioner (GP) to confirm that the participant is still alive. Permission to contact the GP at day 90 will be sought at the time of consent.

## Blood Samples

Blood samples will be taken on two occasions during the trial. Baseline bloods will be taken on day 0 prior to starting G-CSF (or placebo) treatment. A second blood test will be taken on day 5. The research nurse will take the blood tests at the participant’s home when they visit to administer the injection.

Full blood count, platelet studies and CD34 count will be performed on samples by NUH clinical pathology and University of Nottingham staff. No blood samples will be stored. Any blood not utilised will be destroyed.

### Compliance

Compliance will be assessed by recording treatment administration by research nurse on treatment log. Compliance will also be assessed by recording, and returns of residual/unused trial medications. Compliance will be recorded on the case report forms at end of G-CSF or placebo treatment (day 5).

### Accountability for drugs & placebos

The pharmacist will maintain records of the dispensing of the IMP and the research nurse will record administration of the IMP to the participant. Administration details will be recorded on each participants CRF. Unused and partially used supplies will be returned to pharmacy. This will be recorded in the pharmacy study log.

### Management of study drug overdose

No specific antidotes are available. The study drug will be administered by subcutaneous injection by qualified nursing staff so the potential for overdose is not anticipated. The effects of G-CSF overdose have not been established. Discontinuation of G-CSF therapy usually results in a 50% decrease in circulating neutrophils within 1 to 2 days, with a return to normal levels in 1 to 7 days.

### Criteria for terminating trial

The trial may be terminated as a result of a formal or informal interim analysis and based on overwhelming evidence of major safety concerns, new information, or issues with trial conduct (e.g. poor recruitment, loss of resources). Any decision to stop the study prematurely will be based on asymmetric stopping rules^25^, which allow for termination of the study in the event of a negative result otherwise the study will continue until 60 patients have been recruited.

In the event of trial termination, unused drugs will be returned to pharmacy for destruction, and this will be recorded on the pharmacy log, as per unused and partially used supplies.

# STATISTICS

### Methods

As this is a pilot study the analysis will be mainly descriptive. The primary and secondary measures will be compared between participants randomised to G-CSF versus placebo (intention-to-treat) and therapy versus control. No subgroup analyses will be performed.

### Sample size and justification

This is a pilot study assessing feasibility therefore no formal sample size calculation has been performed. Pilot data will be utilised to perform sample size calculation for the definitive trial. The Stroke Trials Unit has experience in delivering clinical stroke trials and we believe recruitment will be achievable. Clinical experience suggests there are a number of stroke participants with residual disability who are not receiving ongoing therapy. From previous experience we anticipate screening 5 participants to recruit one but will collect data on this to help design the definitive trial.

Compliance and losses to follow-up will be monitored but we do not anticipate any problems.

### Assessment of efficacy

Surrogate markers of efficacy:

Haematological: FBC, WCC. PLT and CD34 at Day 5.

Post therapy intervention (day 45) Motor function (RMA); change in dependency (modified Rankin Scale shift); change in disability (change in BI); quality of life (EuroQoL); care giver burden. Change in balance (Berg Balance Scale (BBS). Change in mental state (Mini-Mental State Examination (MMSE). Change in mood (Zung Depression Scale). Change in dependency (Nottingham Extended Activities of Daily Living (NE-ADL).

Three months (day 90): Motor function (RMA); change in dependency (modified Rankin Scale shift); change in disability (change in BI); quality of life (EuroQoL); care giver burden. Change in balance (Berg Balance Scale (BBS). Change in mental state (Mini-Mental State Examination (MMSE). Change in mood (Zung Depression Scale). Change in dependency (Nottingham Extended Activities of Daily Living (NE-ADL).

End follow up (day365): Motor function (RMA); change in dependency (modified Rankin Scale shift); change in disability (change in BI); quality of life (EuroQoL); care giver burden. Change in balance (Berg Balance Scale (BBS). Change in mental state (Mini-Mental State Examination (MMSE). Change in mood (Zung Depression Scale). Change in dependency (Nottingham Extended Activities of Daily Living (NE-ADL).

### Assessment of safety

Serious adverse events, stroke recurrence and infections reported after G-CSF administration (see later for definitions).

### Procedures for missing, unused and spurious data

Missing data will be reported, last observations will not be carried forward for analysis.

### Definition of populations analysed

This is a feasibility study and data will be reported as per allocated treatment groups. The following populations are defined:

Safety set: All randomised participants who receive at least one dose of the study drug.

Full Analysis set: All randomised participants, who take at least one dose of study medication and for whom at least one post-baseline assessment of the primary endpoint is available.

Safety summaries will be performed on the safety set.

# ADVERSE EVENTS

### Definitions

An adverse event is any unfavourable and unintended sign, symptom, syndrome or illness that develops or worsens during the period of observation in the study.

An AE does include a / an:

1. exacerbation of a pre-existing illness.

2. increase in frequency or intensity of a pre-existing episodic event or condition.

3. condition detected or diagnosed after medicinal product administration even though it may have been present prior to the start of the study.

4. continuous persistent disease or symptoms present at baseline that worsen following the start of the study.

An AE does not include a / an:

1. medical or surgical procedure (e.g., surgery, endoscopy, tooth extraction, transfusion); but the condition that lead to the procedure is an AE.

2. pre-existing disease or conditions present or detected at the start of the study that did not worsen.

3. situations where an untoward medical occurrence has not occurred (e.g., hospitalisations for cosmetic elective surgery, social and / or convenience admissions).

4. disease or disorder being studied or sign or symptom associated with the disease or disorder unless more severe than expected for the participant’s condition.

5. overdose of concurrent medication without any signs or symptoms.

A Serious Adverse Event (SAE) is any adverse event occurring following study mandated procedures, having received the IMP or placebo that results in any of the following outcomes:

1. Death

2. A life-threatening adverse event

3. Inpatient hospitalisation or prolongation of existing hospitalisation

4. A disability / incapacity

5. A congenital anomaly in the offspring of a participant

Important medical events that may not result in death, be life-threatening, or require hospitalisation may be considered a serious adverse event when, based upon appropriate medical judgment, they may jeopardize the patient or participant and may require medical or surgical intervention to prevent one of the outcomes listed in this definition.

All adverse events will be assessed for seriousness, expectedness and causality:

A distinction is drawn between serious and severe AEs. Severity is a measure of intensity whereas seriousness is defined using the criteria above. Hence, a severe AE need not necessarily be serious.

### Causality

**Not related or improbable**: a clinical event including laboratory test abnormality with temporal relationship to trial treatment administration which makes a causal relationship incompatible or for which other drugs, chemicals or disease provide a plausible explanation**.** This will be counted as “unrelated” for notification purposes.

**Possible**: a clinical event, including laboratory test abnormality, with temporal relationship to trial treatment administration which makes a causal relationship a reasonable possibility, but which could also be explained by other drugs, chemicals or concurrent disease. This will be counted as “related” for notification purposes.

**Probable**: a clinical event, including laboratory test abnormality, with temporal relationship to trial treatment administration which makes a causal relationship a reasonable possibility, and is unlikely to be due to other drugs, chemicals or concurrent disease. This will be counted as “related” for notification purposes.

**Definite**: a clinical event, including laboratory test abnormality, with temporal relationship to trial treatment administration which makes a causal relationship a reasonable possibility, and which can definitely not be attributed to other causes. This will be counted as “related” for notification purposes.

An AE whose causal relationship to the study IMP is assessed by the Chief Investigator as “possible”, “probable”, or “definite” is an Adverse Drug Reaction.

With regard to the criteria above, medical and scientific judgment shall be used in deciding whether prompt reporting is appropriate in that situation.

### Reporting of adverse events

Participants will be asked to contact the study site immediately in the event of any serious adverse event. All adverse events will be recorded and closely monitored until resolution, stabilisation, or until it has been shown that the study medication or treatment is not the cause. The Chief Investigator shall be informed immediately of any serious adverse events and shall determine seriousness and causality in conjunction with any treating medical practitioners.

In the event of a pregnancy occurring in a trial participant or the partner of a trial participant monitoring shall occur during the pregnancy and after delivery to ascertain any trial related adverse events in the mother or the offspring. Where it is the partner of trial participant consent will be obtained for this observation from both the partner and her medical practitioner.

All serious adverse events will be recorded and reported to the MHRA and REC as part of the annual reports. SUSARs will be reported within the statutory timeframes to the MHRA and REC as stated below. The Chief Investigator shall be responsible for all adverse event reporting.

## SUSARs

**A serious adverse event that is either sudden in its onset, unexpected in its severity and seriousness or not a known side effect of the IMP *and* related or suspected to be related to the IMP is classed as Suspected Unexpected Serious Adverse Reaction and requires expedited reporting as per the clinical trials regulations.**

**All serious adverse events that fall or are suspected to fall within these criteria shall be treated as a SUSAR until deemed otherwise.**

**The event shall be reported immediately of knowledge of its occurrence to the Chief Investigator.**

**The Chief Investigator will:**

- Assess the event for seriousness, expectedness and relatedness to the study IMP
- Take appropriate medical action, which may include halting the trial and inform the Sponsor of such action
- If the event is deemed a SUSAR, shall, within seven days, enter the required data on the MHRA’s eSUSAR web site.
- Shall inform the REC using the reporting form found on the NRES web page within 7 days of knowledge of the event
- Shall, within a further eight days send any follow-up information and reports to the MHRA and REC.
- Make any amendments as required to the study protocol and inform the ethics and regulatory authorities as required

## Trial Treatment Related SAEs

**A serious adverse event that is unexpected in its severity and seriousness *and* deemed directly related to or suspected to be related to the trial treatment but not the IMP shall be reported to the ethics committee that gave a favourable opinion as stated below.**

**The event shall be reported immediately of knowledge of its occurrence to the Chief Investigator.**

**The Chief Investigator will:**

- Assess the event for seriousness, expectedness and relatedness to the trial treatment.
- Take appropriate medical action, which may include halting the trial and inform the Sponsor of such action.
- If the event is deemed related to the trial treatment shall inform the REC using the reporting form found on the NRES web page within 7 days of knowledge of the event.
- Shall, within a further eight days send any follow-up information and reports to the REC.
- Make any amendments as required to the study protocol and inform the REC as required

### Participant removal from the study due to adverse events

Any participant who experiences an adverse event may be withdrawn from the study at the discretion of the Investigator.

# QUALITATIVE SUB STUDY

A qualitative study of the feasibility and acceptability of participation in the STEMS3 trial: The participants, non-participants, carers and clinicians perspective.

Aim

This qualitative study is designed to compliment the data collected in STEMS3 and capture the hidden data that will influence the design and methodology of a trial of this type in the future. It will aim to elicit data about the recruitment process, enrollment and the trial experience through the use of semi-structured interviews.

Objectives

1. To gain an understanding of what it means to participants to take part in STEMS3 and how this influences recruitment, decision making and trial experience.
2. To gain an understanding of what clinicians feel about STEMS3 and the barriers and motivations to participant identification.

Design

Semi-structured interviews.

Participants

Subjects for the qualitative sub-study will be STEMS3 participants (participants), eligible for STEMS3 but declined to take part (non-participant), carers of participants and non-participants (carers) or clinicians involved in recruitment (clinicians). All participants must have no cognitive impairments or language difficulties that would prevent them taking part in an interview.

Sampling

Subjects will be sampled purposively from each sub-group. Ideally this will occur until theoretical saturation has been reached but it is possible that sampling will be limited by the time and funding resources available. It is likely that approximately 10 participants will be sampled with equal representation of those that received the physiotherapy intervention (n=5) and those that did not (n=5). Within this group it is planned that approximately 50% will have received G-CSF and 50% will have received placebo injection; the subject will remain blinded to this allocation. Approximately 5 carers, non-participants and clinicians will be sampled.

Data collection

Data will be collected following iterative and emergent principles using semi-structured interviews. This will allow the objectives to be explored guided by topics that have emerged anecdotally from STEMS3 to date, and allow any new topics to be explored. Semi-structured interviews are conducted using open ended questions that define the area and may then be followed with questions that diverge to pursue a response or idea in more detail. This approach has the advantage of being flexible to allow any line of enquiry that may arise to be pursued; this will maximize the validity. The initial topic guide was formed based on a broad list of topics which have arisen and provoked discussion during STEMS3.

Topic guide

Participants

- Can you tell me what you understand about STEMS3?
- How did you find out about STEMS3? How was the information?
- What made you decide you wanted to take part?
- What was your experience of being part of the trial?
- How do you feel about using a drug with rehabilitation?
- What did it mean to you to take part in the trial?
- Did you feel that the trial benefitted you in any way?

Non-participants

- Can you tell me what you understand about the trial?
- How did you find out about the trial? How was the information?
- What made you decide you didn’t want to take part?

Carers

- Can you tell me what you understand about the trial?
- How did you find out about the trial? How was the information?
- Were you involved in the participant/ non-participants decision to take part?
- What did it mean to you for the participant to take part?
- Were there any benefits to you and the participant in taking part?

Clinicians involved in identifying potential STEMS3 participants

- Can you tell me what you understand about the trial?
- What do you think about the trial?
- Can you tell me how you decided who would meet the inclusion criteria?
- How did you feel about the recruitment process?
- Did you have any concerns about the ethics of the trial?

The interview guide will be refined after the first interviews within each of the subject groups depending on the data elicited with the purpose of exploring new issues as they are raised.

A neutral interviewer, skilled in semi-structured interview techniques, will conduct interviews. It is hoped that this will reduce bias and allow the participants to speak openly and honestly about their experiences. The subject will be given a choice of interview venue. This should be a quiet room where interruptions are unlikely. Interviews will be tape recorded and transcribed verbatim by the research therapist, coded and analyzed with the aid of qualitative data analysis computer package NVivo. Field notes will also be taken during the interview to enrich the data. Data will be stored in a password protected folder on the investigators University computer. Audio recordings will stored securely in an archive accessible to the investigators and destroyed 7 years after the last publication arising from the sub study. These procedures are in accordance with the University’s research policy.

Data Analysis

Prior to analysis the researcher will become familiar with tape recordings, transcripts, field notes and any other data collected. Data will be organized by topic and coded by theme. This process will take place concurrently with the interviews as the data can inform and improve the research process. The coded data will then be analysed and interpreted. It is likely that themes will emerge from the data and through constant comparison, the differences and similarities between subsequent interviews can be examined. To ensure reliability, the analysis of data from the two researchers will be compared and debated until an agreed conclusion is made.

# ETHICAL AND REGULATORY ASPECTS

## ETHICS COMMITTEE AND REGULATORY APPROVALS

The trial will not be initiated before the protocol, informed consent forms and participant and GP information sheets have received approval / favourable opinion from the Medicines and Healthcare products Regulatory Agency (MHRA), Research Ethics Committee (REC), and the respective National Health Service (NHS) Research & Development (R&D) department. Should a protocol amendment be made that requires REC approval, the changes in the protocol will not be instituted until the amendment and revised informed consent forms and participant and GP information sheets (if appropriate) have been reviewed and received approval / favourable opinion from the REC and R&D departments. A protocol amendment intended to eliminate an apparent immediate hazard to participants may be implemented immediately providing that the MHRA, R&D and REC are notified as soon as possible and an approval is requested. Minor protocol amendments only for logistical or administrative changes may be implemented immediately; and the REC will be informed.

The trial will be conducted in accordance with the ethical principles that have their origin in the Declaration of Helsinki, 1996; the principles of Good Clinical Practice, in accordance with the Medicines for Human Use Regulations, Statutory Instrument 2004, 1031 and its subsequent amendments and the Department of Health Research Governance Framework for Health and Social care, 2005.

## INFORMED CONSENT AND PARTICIPANT INFORMATION

The process for obtaining participant informed consent will be in accordance with the REC guidance, and Good Clinical Practice (GCP) and any other regulatory requirements that might be introduced. The investigator or their nominee and the participant shall both sign and date the Consent Form before the person can participate in the study.

The participant will receive a copy of the signed and dated forms and the original will be retained in the Trial Master File. A second copy will be filed in the participant’s medical notes and a signed and dated note made in the notes that informed consent was obtained for the trial.

The decision regarding participation in the study is entirely voluntary. The investigator or their nominee shall emphasize to them that consent regarding study participation may be withdrawn at any time without penalty or affecting the quality or quantity of their future medical care, or loss of benefits to which the participant is otherwise entitled. No trial-specific interventions will be done before informed consent has been obtained.

The investigator will inform the participant of any relevant information that becomes available during the course of the study, and will discuss with them, whether they wish to continue with the study. If applicable they will be asked to sign revised consent forms.

If the Informed Consent Form is amended during the study, the investigator shall follow all applicable regulatory requirements pertaining to approval of the amended Informed Consent Form by the REC and use of the amended form (including for ongoing participants).

## RECORDS

### Drug accountability

The IMP will be prescibed and dispensed on an individual participant basis, in 5 day treatment packs. The pharmacist will maintain records of the distribution of IMP and the research nurse will record administration of the IMP to the participant. The research nurse will return any unused IMP to NUH trials pharmacy.

The investigator and the local site pharmacist shall maintain records of the study drug’s delivery to the pharmacy, an inventory at the site, the distribution to each participant, and the return to the pharmacy of unused study drugs. These records will include dates, quantities received, batch / serial numbers, expiration dates, and the unique code numbers (participant trial number) assigned to the trial participant. Investigators and /or the local site pharmacists will maintain records that document adequately that the participants were provided with the correct study medication. These records will be part of each participant’s Case Report Form (CRF). All study medication received by the pharmacy shall be accounted for.

### Case Report Forms

Each participant will be assigned a trial identity code number, allocated at randomisation if appropriate, for use on CRFs other trial documents and the electronic database. The documents and database will also use their initials (of first and last names separated by a hyphen or a middle name initial when available) and date of birth (dd/mm/yy).

CRFs will be treated as confidential documents and held securely in accordance with regulations. The investigator will make a separate confidential record of the participant’s name, date of birth, local hospital number or NHS number, and Participant Trial Number (the Trial Recruitment Log), to permit identification of all participants enrolled in the trial in accordance with regulatory requirements and for follow-up as required.

CRFs shall be restricted to those personnel approved by the Chief or local Principal Investigator and recorded on the ‘Trial Delegation Log.’

All paper forms shall be filled in using black ballpoint pen. Errors shall be lined out but not obliterated by using correction fluid and the correction inserted, initialled and dated.

The Chief or local Principal Investigator shall sign a declaration ensuring accuracy of data recorded in the CRF.

### Source documents

Source documents shall be filed at the investigator’s site and may include but are not limited to, consent forms, current medical records, laboratory results and pharmacy records. A CRF may also completely serve as its own source data. Only trial staff as listed on the Delegation Log shall have access to trial documentation other than the regulatory requirements listed below.

### Direct access to source data / documents

The CRF and all source documents, including progress notes and copies of laboratory and medical test results shall made be available at all times for review by the Chief Investigator, Sponsor’s designee and inspection by relevant regulatory authorities (e.g., MHRA).

## DATA PROTECTION

All trial staff and investigators will endeavour to protect the rights of the trial’s participants to privacy and informed consent, and will adhere to the Data Protection Act, 1998. The CRF will only collect the minimum required information for the purposes of the trial. CRFs will be held securely, in a locked room, or locked cupboard or cabinet. Access to the information will be limited to the trial staff and investigators and relevant regulatory authorities (see above). Computer held data including the trial database will be held securely and password protected. All data will be stored on a secure dedicated web server. Access will be restricted by user identifiers and passwords (encrypted using a one way encryption method).

Information about the trial in the participant’s medical records / hospital notes will be treated confidentially in the same way as all other confidential medical information.

Electronic data will be backed up every 24 hours to both local and remote media in encrypted format.

# QUALITY ASSURANCE & AUDIT

## INSURANCE AND INDEMNITY

Insurance and indemnity for trial participants and trial staff is covered within the NHS Indemnity Arrangements for clinical negligence claims in the NHS, issued under cover of HSG (96)48. There are no special compensation arrangements, but trial participants may have recourse through the NHS complaints procedures.

The University of Nottingham has taken out an insurance policy to provide indemnity in the event of a successful litigious claim for proven non-negligent harm.

## TRIAL CONDUCT

Trial conduct will be subject to systems audit of the Trial Master File for inclusion of essential documents; permissions to conduct the trial; Trial Delegation Log; CVs of trial staff and training received; local document control procedures; consent procedures and recruitment logs; adherence to procedures defined in the protocol (e.g. inclusion / exclusion criteria, correct randomisation, timeliness of visits); adverse event recording and reporting; drug accountability, pharmacy records and equipment calibration logs.

The Trial Coordinator, or where required, a nominated designee of the Sponsor, shall carry out a site systems audit at least yearly and an audit report shall be made to the Trial Steering Committee.

## TRIAL DATA

Monitoring of trial data shall include confirmation of informed consent; source data verification; data storage and data transfer procedures; local quality control checks and procedures, back-up and disaster recovery of any local databases and validation of data manipulation. The Trial Coordinator, or where required, a nominated designee of the Sponsor, shall carry out monitoring of trial data as an ongoing activity.

Entries on CRFs will be verified by inspection against the source data. A sample of CRFs (10% or as per the trial risk assessment) will be checked on a regular basis for verification of all entries made. In addition the subsequent capture of the data on the trial database will be checked. Where corrections are required these will carry a full audit trail and justification.

Trial data and evidence of monitoring and systems audits will be made available for inspection by the regulatory authority as required.

## RECORD RETENTION AND ARCHIVING

In compliance with the ICH/GCP guidelines, regulations and in accordance with the University of Nottingham Code of Research Conduct and Research Ethics, the Chief or local Principal Investigator will maintain all records and documents regarding the conduct of the study. These will be retained for at least 7 years or for longer if required. If the responsible investigator is no longer able to maintain the study records, a second person will be nominated to take over this responsibility.

The Trial Master File and trial documents held by the Chief Investigator on behalf of the Sponsor shall be finally archived at secure archive facilities at the University of Nottingham. This archive shall include all trial databases and associated meta-data encryption codes.

## DISCONTINUATION OF THE TRIAL BY THE SPONSOR

The Sponsor reserves the right to discontinue this trial at any time for failure to meet expected enrolment goals, for safety or any other administrative reasons. The Sponsor shall take advice from the Trial Steering Committee and Data Monitoring Committee as appropriate in making this decision.

## STATEMENT OF CONFIDENTIALITY

Individual participant medical information obtained as a result of this study are considered confidential and disclosure to third parties is prohibited with the exceptions noted above.

Participant confidentiality will be further ensured by utilising identification code numbers to correspond to treatment data in the computer files.

Such medical information may be given to the participant’s medical team and all appropriate medical personnel responsible for the participant’s welfare.

Data generated as a result of this trial will be available for inspection on request by the participating physicians, the University of Nottingham representatives, the REC, local R&D Departments and the regulatory authorities.

# PUBLICATION AND DISSEMINATION POLICY

The pilot trial results will be published in an academic journal. The focus of that article will be to discuss the feasibility or otherwise of a definitive trial to test the effectiveness and safety of G-CSF and therapy in chronic stroke. In particular, the feasability of delivering treatments in participants within the community

While this pilot study is aimed at assesing the feasability of a definitive trial, and not powered to detect efficacy, the results, even of 60 will have some clincal importance. Data from the trial (as with our first two) will be shared with the Cochrane Collaboration systematic review on colony stimulating factors.

We will take steps to ensure that besides collecting the data we need for trial feasibility that participants also collect data on trial efficacy, for this pupose. Such data will be in the form that the Cochrane review has used previously.

The Nottingham Stroke Consumer Group will give information about the pilot study on the support group website. When the pilot study is complete we will post summary findings there. If the main trial proves to be feasible we will use the support group to help publicise it and ensure it is sucessful.

Findings will also be presented at conferences such as UK Stroke Forum and Society for Research in Rehabilitation.

# USER AND PUBLIC INVOLVEMENT

The project and protocol was discussed at the Nottingham Stroke Consumer Group meeting on July 27^th^ 2009. The group reviewed the trial design, made some suggestions regarding design and were highly supportive of the project.

A member of the Stroke Consumer Group will be involved in the subsequent designing of the definitive study and a lay member of the definitive Trial Steering Committee.

The Stroke Consumer Group will aslo help with dissemination of the results via the user group website.

# STUDY FINANCES

### Funding source

This study is funded by National Institute for Health Research - Research for Patient Benefit ref. PB-PG-0909-19113.

### Participant stipends and payments

Participants will not be paid to participate in the trial. Travel expenses will be offered for any hospital visits in excess of usual care .

# SIGNATURE PAGES

Signatories to Protocol:

**Chief Investigator:** (name)__________________________________

Signature:__________________________________

Date: ___________

**Trial Statistician**: (name)__________________________________

Signature:__________________________________

Date: ___________

**Trial Pharmacist**: (name)__________________________________

Signature:__________________________________

Date: ___________

# REFERENCES

1. Nudo RJ. Functional and structural plasticity in motor cortex: implications for stroke recovery. Phys Med Rehabil Clin N Am 2003; 14: 57–76.

2. Sprigg N, Bath PMW. Pharmacological enhancement of recovery from stroke. Current Medical Literature: Stroke Review. 2005;8:33-39

3. Cavallaro AM, Lilleby K, Majolino I, Storb R, Appelbaum FR, Rowley SD, Bensinger WI. Three to six year follow-up of normal donors who received recombinant human granulocyte colony-stimulating factor. Bone Marrow Transplantation. 2000;25:85-89

4. Corti S, Locatelli F, Strazzer S, Salani S, Del Bo R, Soligo D, Bossolasco P, Bresolin N, 5. Scarlato G, Comi GP. Modulated generation of neuronal cells from bone marrow by expansion and mobilization of circulating stem cells with in vivo cytokine treatment. Experimental Neurology. 2002;177:443-452

6. Gibson CL, Bath PMW, Murphy SP. G-csf reduces infarct volume and improves functional outcome after transient focal cerebral ischemia in mice. Journal of Cerebral Blood Flow and Metabolism. 2005;25:431-439

7. Schneider A, Kruger IC, Steigleder IT, Weber D, Pitzer C, Laage R, Aronowski IJ, Maurer MH, Gassler N, Mier W, Hasselblatt M, Kollmar R, Schwab S, Sommer C, Bach A, Kuhn HG, Schabitz WR. The hematopoietic factor g-csf is a neuronal ligand that counteracts programmed cell death and drives neurogenesis. Journal of Clinical Investigation. 2005;115:2083 - 2098

8. Shyu WC, Lin SZ, Yang HI, Tzeng YS, Pang CY, Yen PS, Li H. Functional recovery of stroke rats induced by granulocyte colony-stimulating factor- stimulated cells. Circulation. 2004;110:1847-1854

9. England TJ, Gibson CL, Bath PMW. Granulocyte-colony stimulating factor in experimental stroke and its effects on infarct size and functional outcome: A systematic review. Brain Research Reviews. 2009;62:71-82

10. Park HK, Kon Chu K, Lee ST, Jung KH, Kim EH, Lee KB, Song YM, Jeong SW, Kim M, Roh JK. Granulocyte colony-stimulating factor induces sensorimotor recovery in intracerebral hemorrhage. Brain Research. 2005;1041:125-131

11. Zhang J, Deng M, Zhang Y, Sui W, Wang L, Sun A, Song H, Lu M, Fan D. A short-term assessment of recombinant human granulocyte-stimulating factor (rhg-csf) in treatment of acute cerebral infarction (abstract). Cerebrovascular diseases. 2006;21(suppl 4):143

12. Shyu WC, Lin SZ, Lee CC, Liu DD, Li H. Granulocyte colony-stimulating factor for acute ischemic stroke: A randomized controlled trial. Canadian Medical Association Journal. 2006;174:927-933

13. Sprigg N, Bath P, L. Z, Willmot M, LJ. G, Walker M, Dennis M, Russell M. Granulocyte-colony stimulating factor mobilises bone marrow stem cells in patients with sub-acute ischaemic stroke: The 'stem cell trial of recovery enhancement after stroke' (stems) pilot randomised controlled trial. Stroke. 2006;37:2979-2983

14. Bath P, Sprigg N. Colony stimulating factors (including erythropoietin, granulocyte-colony stimulating factor and analogues) for stroke. Cochrane Database Syst Rev. 2007;Issue 2

15. England TJ, Bath PMW. 'stem cell trial of recovery enhancement after stroke 2' (stems2): Pilot randomised placebo-controlled trial of granulocyte-colony stimulating factor in mobilising bone marrow stem cells in sub-acute stroke. 2009

16. Schabitz WR. Axis2 (g-csf) for the treatment of ischemic stroke. http://www.strokecenter.org/trials. 2009

17. Zhao L-R, Berra HH, Duan W-M, Singhal S, Mehta J, Apkarian A, Vania., Kessler J, A. Beneficial effects of hematopoietic growth factor therapy in chronic ischemic stroke in rats. Stroke. 2007;38:2804-2811

18. Chun-Shu P, Maria E, Gonzalez-Toledo., Yue-Qiang X, Wei-Ming D, Satoshi T, D N, Granger., Roger EK, Li-Ru Z. The role of stem cell factor and granulocyte-colony

stimulating factor in brain repair during chronic stroke Journal of Cerebral Blood Flow & Metabolism 2009;29:759-770

19. N. Byl, J. Roderick, O. Mohamed, M. Hanny, J. Kotler, A. Smith, M. Tang, Abrams G. Effectiveness of sensory and motor rehabilitation of the upper limb following the principles of neuroplasticity: Patients stable poststroke. Neurorehabil Neural Repair. 2003;17:176

20. N. N. Byl EAP, and G. M. Abrams. Functional outcomes can vary by dose: Learning-based sensorimotor training for patients stable poststroke. Neurorehabil Neural Repair. 2008;22:494-504

21. Kwakkel G, Wagenaar RC, Twisk JWR, Lankhorst GJ, Koetsier JC. Intensity of leg and arm training after primary middle-cerebral-artery stroke: A randomised trial. Lancet. 1999;354:191-196

22. Legg L, Langhorne P, Outpatient Service Trialists. Rehabilitation therapy services for stroke patients living at home: Systematic review of randomised trials. Lancet. 2004;363:352-356

23. Aziz NA, Leonardi-Bee J, Phillips MF, Gladman J, Legg LA, Walker M. Therapy-based rehabilitation services for patients living at home more than one year after stroke. 2008, issue 2. Cochrane Database of Systematic Reviews 2008

24.Feeney DM, Gonzalez A, Law WA. Amphetamine, haloperidol, and experience interact to affect rate of recovery after motor cortex injury. Science. 1982;217:855-857

25. Demets. D. L. & Ware, J. H. (1980). Group sequential methods for clinical trials with a one-sidedhypothesis. Biometrika 67, 651 60.
